# Niosomes encapsulated in biohydrogels for tunable delivery of phytoalexin resveratrol†

**DOI:** 10.1039/c8ra09655d

**Published:** 2019-03-08

**Authors:** Noelia D. Machado, Mariana A. Fernández, Marleen Häring, César Saldías, David Díaz Díaz

**Affiliations:** Instituto de Investigaciones en Físico-Química de Córdoba (INFIQC-CONICET), Departamento de Química Orgánica, Facultad de Ciencias Químicas, Universidad Nacional de Córdoba, Ciudad Universitaria X5000HUA Córdoba Argentina; Institute of Organic Chemistry, University of Regensburg Universitätstrasse. 31 93040 Regensburg Germany David.Diaz@chemie.uni-regensburg.de; Departamento de Química Física, Facultad de Química, Pontificia Universidad Católica de Chile Macul Santiago Chile; Instituto de Productos Naturales y Agrobiología del CSIC Avda. Astrofísico Francisco Sánchez 3 38206 La Laguna Tenerife Spain d.diaz.diaz@ipna.csic.es

## Abstract

A series of biohydrogels based on mixtures of kappa-carrageenan (κ-carrageenan, κ-C) and gelatin were evaluated as potential soft delivery vehicles for the encapsulation and subsequent release of non-ionic surfactant vesicles (niosomes) loaded with resveratrol (RSV). The niosomes were prepared using a mixture of amphiphilic lipids Tween 80 and Span 80 in water. The results showed that RSV-niosomes did not significantly affect the hydrogelation properties of the biopolymer mixture. Moreover, *in vitro* drug release experiments from biohydrogels containing RSV-niosomes were successfully carried out under simulated gastrointestinal conditions. The RSV-niosomal liberation profiles from hydrogels were fitted using first order kinetics, Higuchi, Korsmeyer–Peppas and Weibull drug release models, showing the prevalence of diffusion mechanisms in each case. In addition, the RSV release was easily tuned by adjusting the total concentration of κ-C : gelatin. Interestingly, the niosomal-hydrogel system was also found to prevent the *trans*-to-*cis* photoisomerization of RSV.

## Abstract 2

A series of biohydrogels were evaluated as potential soft delivery vehicles for the encapsulation and subsequent release of niosomes loaded with resveratrol (RSV).

## Graphical abstract

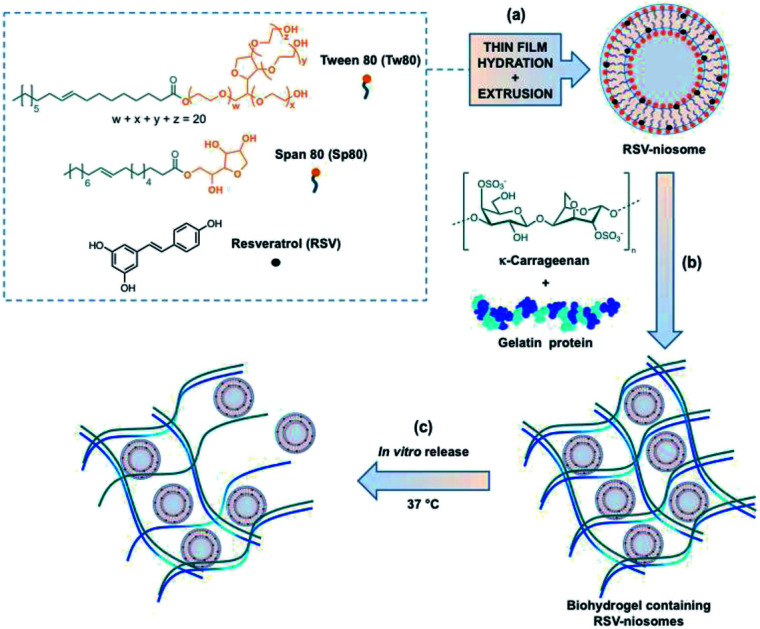

## 1. Introduction

Most drugs and nutraceutical compounds, such as antioxidants and vitamins, are highly lipophilic molecules that require encapsulation into different delivery systems to circumvent a series of limitations. These are primarily due to physicochemical instability of these molecules under physiological conditions and/or poor biodisponibility.^1^ In particularly, resveratrol (RSV) is a nutraceutical compound that belongs to the stilbene family of phytoalexins, and it is mainly found in grape seeds and skin extracts. This natural phenolic compound is produced by several plants either in response to injury or when the plant is under attack by pathogens such as bacteria or fungi. Numerous studies have demonstrated that RSV possesses a wide range of biological properties such as cardioprotection,^2,3^ anti-inflammatory and antioxidant activities,^4–7^ among others. However, the application of RSV in pharmaceutical and food industries is usually limited owing to its low water solubility, and rapid UV-induced isomerization to the inactive biologically isomer (*cis* isomer).^8–10^ The low oral bioavailability of RSV is a product of susceptibility to sulfation and glucuronidation reactions during phase II of metabolism in the gastrointestinal tract.^11^

Within this context, many drug delivery systems (DDS) have been developed during the last decade to control retention, stability, solubility and release of bioactive compounds.^12–15^ Among numerous DDS, vesicles are colloidal particles formed by concentric bilayers, composed of amphiphilic molecules, that surround an aqueous inner phase. Niosomes, a specific class of vesicles formed by non-ionic surfactants, provides a versatile chemical environment for the encapsulation of either hydrophilic or hydrophobic compounds.^16,17^ Niosomes are also cheaper than other structurally related vesicles such as liposomes, and do not require special storage conditions.

Another broadly used DDS are hydrogels, which consist in 3D solid networks formed by chemical or physical crosslinking of polymers that can immobilize high amounts of water or biological fluids into the interstices.^18^ The use of hydrogels derived from natural polymers (*i.e.*, biohydrogels) is particularly interesting due to their degradability, biocompatibility, good mechanical properties, inyectability, non-immunogenicity and abundance in nature.^19^ It has been demonstrated that drugs and nutraceuticals delivery systems based on hydrogels allow to enhance their bioavailability, increase the versatility of administration routes, and improve their residence time in the gastrointestinal tract.^20^ Moreover, a commun strategy for the encapsulation and tunable release of active molecules is the use of hydrogels made of mixtures of polymers. The release rate depends on the composition and chemical structure of the biopolymers, among other factors.^21^ Studies employing this kind of systems have been focused in the field of pharmacotherapeutics for the treatment of cancer,^22–25^ acne,^26^ pain,^27^ psoriasis,^28^ ocular hypertension,^29,30^ alopecia,^31^ vitiligo,^32^ among other diseases.^33–36^

Among different biopolymers commonly used for the preparation of hydrogels, gelatin and carrageenans have been extensively studied and used in numerous applications. Gelatin is a mixture of peptides and proteins produced by partial and irreversible hydrolysis of collagen extracted from bones, skin, and connective tissues of animals. This biopolymer is widely used in food, cosmetic, and pharmaceutical industries due to its non-cytotoxic, non-immunogenic, biodegradability, and hemocompatibility properties.^37,38^ On the other hand, carrageenans are a family of high-molecular-weight linear sulfated polysaccharides that are extracted from red edible seaweeds. They are widely used in the food industry, for their thickening, gelling, and stabilizing properties, as well as for many biomedical applications.^39,40^ From a chemical point of view, all carrageenans are made up of repeating galactose units and 3,6-anhydrogalactose, both sulfated and non-sulfated. The units are connected by alternating α-1,3 and β-1,4 glycosidic linkages, allowing for the formation of curling helical structures. The number and position of the ester sulfate groups on the repeating galactose units determine the properties of different types of carrageenans (*i.e.*, kappa, iota, and lambda). The solubility temperature of the carrageenan decreases with the amount of sulfate groups, which produce lower strength gels or contribute to gel inhibition. In particular, kappa-carrageenan (κ-carrageenan, κ-C) contains one sulfate group per disaccharide and forms strong gels, whereas iota-carrageenan forms soft gels and lambda-carrageenan does not form gels.

There are reports about the incorporation of RSV in vesicles with the idea to fortify some foods^41^ modulating the antioxidant delivery rate. However it is known that liposomes, niosomes and other lipid particles may suffer stability problems *in vivo* depending on the form of administration. The inclusion of vesicles inside a hydrogel matrix has generated promising hybrid materials^42,43^ that could help to improve the efficacy of the formulations. In this way, it could be possible to protect colloidal particles as well as to reduce undesirable burst release effects of the loaded compound.^44^ Particularly, protection of RSV from rapid metabolization in the gastrointestinal tract could be one possible strategy to increase its bioavailability.^45^

In this work, we have explored the incorporation of RSV-loaded niosomes within hydrogels made of a mixture of κ-C and gelatin protein at different concentrations. The stability of the RSV-niosomes inside the hydrogel, and the photoprotection of RSV against isomerization have been studied. Furthermore, *in vitro* release kinetics of the niosomal formulation from these hydrogels was also investigated and adjusted to several mathematical models. To the best of our knowledge, the use of κ-C : gelatin hydrogel systems to encapsulate niosomes for the controlled delivery of RSV has not yet been reported.

## 2. Experimental section

### 2.1. Materials

All reagents were used as received from commercial suppliers without further purification. Tween 80 (Tw80, oleic acid 70%) was purchased from Riedel-de Haën and Span 80 (Sp80, oleic acid ≤ 60%) from Fluka. κ-carrageenan (κ-C) and gelatin (75% protein) from bovine skin were purchased from Sigma-Aldrich. *trans*-Resveratrol (RSV, > 98%) was purchased from Cayman Chemical Company. Sodium hydroxide, potassium chloride, and potassium dihydrogen phosphate were purchased from Emsure® ISO. 1 N HCl solution was obtained from Sigma Aldrich, absolute EtOH for analysis from Labchem® International, and MeOH for liquid chromatography from Merck. Millipore water purified was used for all the experiments.

### 2.2. General procedures

#### 2.2.1. Preparation of RSV-niosomes

Niosomes were prepared in an equimolar mixture of Tw80 and Sp80 in water (working concentration = 10 mM) using the thin film hydration method followed by extrusion through a 100 nm membrane. Briefly, stock solutions of Tw80, Sp80 and RSV in EtOH were prepared. Appropriate aliquots of non-ionic surfactants and RSV solutions (to obtain a final concentration of 10 mM and 0.22 mM, respectively) were added in a round bottom flask. Then, EtOH was completely removed under vacuum pressure at 40 °C and 100 rpm, using a Heidolph rotary evaporator equipped with a vacuum control box ILMVAC. After the dried thin film was formed, MilliQ water (10 mL) was added and the mixture was stirred at 250 rpm and heated at 60 °C using a water bath during 30 min (Heidolph MR Hei-Standard agitator equipped with a temperature controller EKT Hei-Con). Subsequently, RSV-niosomes were separated from free RSV using size exclusion gel chromatography (14.5 × 50 mm Disposable Desalting Column with Sephadex G-25 resin, GE Healthcare Life Sciences). The final suspension was extruded 21 times through a 100 nm pore size polycarbonate membrane using a manual mini extruder from Avanti Polar Lipids, Inc. Extruded RSV-niosomes were used for the further analysis. In order to quantify the amount of RSV inside the niosomes, a sample (0.1 mL) was diluted in MeOH (1 mL) and the absorbance at 305 nm was recorded on a Varian Cary BIO 50 UV/VIS/NIR Spectrometer. The RSV concentration was determined *via* interpolation from a separately calibration curve.

#### 2.2.2. Preparation of (RSV-niosomes)-containing hydrogel

Typically, a mixture of κ-C and gelatin (1 : 1 mass ratio, 4% w/v) was placed into a screw-capped glass vial (4 cm length × 1 cm diameter) and gently dissolved in RSV-niosomes (1 mL) at 45 °C. The mixture was cooled down to RT affording the corresponding (RSV-niosomes)-containing hydrogel. Control hydrogels were prepared using MilliQ water (1 mL) in the absence of RSV-niosomes following the same procedure (ESI, Fig. S1†). The encapsulation efficiency (EE) of the hybrid materials was calculated by the percentage ratio between the remaining amount of RSV in the receptor phase and the initial RSV amount (starting material) at *t* = 0 [eqn (1)].1
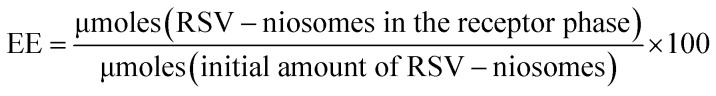


#### 2.2.3. Stability of RSV-niosomes entrapped within hydrogels

RSV niosomes were obtained in water as described above. Subsequently, a solution of the vesicles (1 mL) was carefully added to a mixture of κ-C and gelatin (1 : 1, mass ratio). After polymer swelling, water (3 mL) was added as receptor phase. Hydrogel was incubated at 37 °C for 24 h until hydrogel degradation. Then, fractions of released RSV-niosomes (0.1 mL) were diluted in water (1 mL), and filtered through a syringe filter of 0.45 μm pore size. Hydrodynamic diameter and polydispersity index (PDI) of the vesicles were measured at 25 °C using a Zetasizer Nano ZS (Malvern Instrument).

#### 2.2.4. Field emission scanning electron microscopy (FE-SEM)

FE-SEM images of the bulk xerogels were obtained with a Zeiss Merlin, Field Emission Scanning Electron Microscope operated at an accelerating voltage of 10 kV. Samples were prepared by freeze-drying as following: an Eppendorf tube containing the corresponding hydrogel (volume = 2 mL) was frozen in liquid nitrogen or dry ice/acetone and the solvent was immediately evaporated under reduced pressure (0.6 mmHg) for 2 days at RT. The obtained solid was placed on top of a tin plate and shielded with Pt (40 mA during 30–60 s; film thickness = 5–10 nm).

#### 2.2.5. Stability of *trans*-RSV under UV light irradiation

Solutions of RSV in ethanol, RSV-niosomes and hydrogels containing RSV-niosomes were exposed to UV light irradiation (365 nm, 1 W) during 1 h to induce *trans*-to-*cis* isomerization. After this time, the amount of remaining *trans*-RSV in the samples was measured by chromatography (VWR-Hitachi LaChrome Elite HPLC). The system was equipped with a UV-vis detector (L-2455 LaChrome Elite diode array detector). The analytical column was a Hibar® 250-4Purospher® STAR RP-18 endcapped (5 μm particle size) (Merck). The mobile phase consisted of a mixture of (A) 100% MilliQ water and (B) 100% of MeOH with gradient elution at a flow rate of 0.8 mL min^−1^, following the procedure previously reported.^41^ A wavelength of 305 nm was used for the UV-vis detector. The RSV concentration was determined *via* interpolation from a separately constructed calibration curve. The ratio between the amount of *trans*-RSV remaining in the sample after and before UV light exposure was calculated.

#### 2.2.6. *In vitro* release experiments

Release experiments were carried out under simulated gastrointestinal conditions at 37 °C. A buffer solution at pH 1.2 was prepared using a solution of HCl (0.085 M) and KCl (0.050 M) to achieve gastric conditions, whereas a buffer solution at pH 6.8 was prepared with NaOH (0.022 M) and KH_2_PO_4_ (0.050 M) to imitate intestinal conditions. For the experiments, buffer solution (1 mL) was added to the hydrogels (1 mL volume) placed into a screw-capped glass vial (4 cm length × 1 cm diameter). At specific time intervals (*i.e.*, every 15 min at pH 1.2 and every 1 h at pH 6.8), aliquots (1 mL) were carefully removed for subsequent UV-vis spectrophotometric analysis. The removed volume was replaced with fresh buffer solution (1 mL). The absorbance of each aliquot (1 mL) was measured by using the maximum absorbance wavelength of 305 nm. The cumulative RSV release was obtained by calculating the total amount detected in the aliquots [eqn (2)]. The RSV concentration was determined *via* interpolation from a separately constructed calibration curve.2
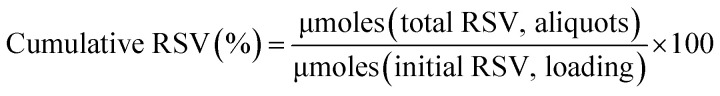


### 2.3. Oscillatory rheological measurements

Oscillatory rheological measurements were carried out in an AR 2000 Advanced rheometer (TA Instruments) equipped with a Julabo C cooling system. A stainless steel plain-plate geometry (20 mm), a 1000 μm gap setting and a torque setting of 40 000 dynes per cm^2^ at 25 °C were used for rheological measurements. After obtaining biohydrogels both in the absence and in the presence of niosomes (total gel volume = 2 mL), the following experiments were carried out: (1) Dynamic Frequency Sweep (DFS) measurements to monitor the variation of *G*′ and *G*′′ with frequency (*i.e.*, from 0.1 to 10 Hz at 0.1% strain); (2) Dynamic Strain Sweep (DSS) measurements to monitor the variation of *G*′ and *G*′′ with strain (*i.e.*, from 0.01 to 100%); and (3) Dynamic Time Sweep (DTS) experiments to monitor the variation of *G*′ and *G*′′ over time maintaining both, the strain and frequency values constant, within the linear viscoelastic regime (*i.e.*, strain = 0.1% strain; frequency = 1 Hz).

Thixotropy was determined by a 3-loop test involving the application of a low shear strain (0.1%) at 1 Hz frequency for 5 min, followed by an increase of the shear strain to *ca.* 50% for 2 min, and final return to the initial shear strain for 10 min.

### 2.4. Mathematical models

Release curves were fitted according to first order kinetics [eqn (3)], Higuchi [eqn (4)], Korsmeyer–Peppas [eqn (5)] and Weibull [eqn (6)] empirical equations, where *M*_*t*_ and *M*_∞_ correspond to the cumulative and the maximal amounts of RSV released at time *t*, respectively. In first order kinetics model, the natural logarithm of the fraction of drug release is proportional to the time, being *k* a rate constant. In the case of Higuchi model, the fraction of drug released is proportional to the square root of time, and *K* is a constant related to the formulation. In Korsmeyer–Peppas model, *k* is a rate constant and *n* is the exponent that characterizes the release mechanism (*e.g.*, *n* = 0.5 corresponds to a Fickian diffusion mechanism, whereas 0.5 < *n* < 1 is typical for non-Fickian diffusions). In the Weibull model, *b* is a parameter that describes the drug diffusional mechanism. Specifically, Fickian diffusion is the main mechanism when *b* ≤ 0.75, while values between 0.75 < *b* < 1 correspond to a diffusion mechanism together with a potential contribution of other complex release processes.3
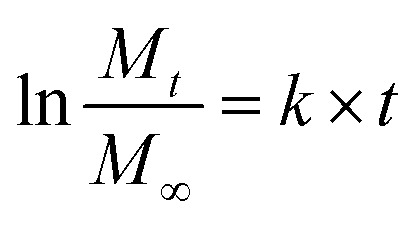
4
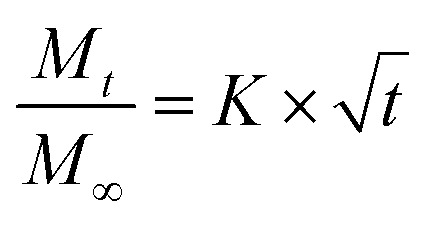
5
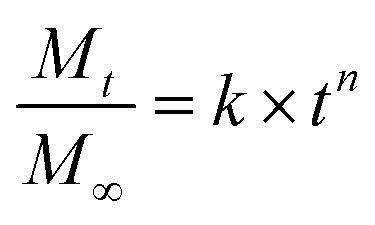
6
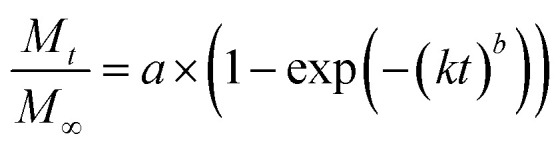


## 3. Results and discussion

### 3.1. Synthesis and characterization of hydrogels containing RSV-niosomes

**Fig. 1:**
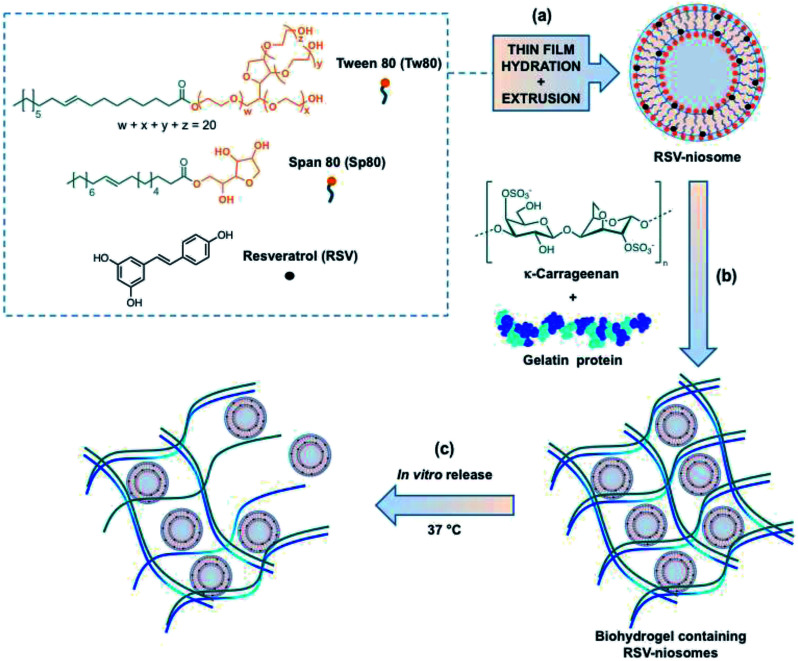
Schematic illustration of the study carried out in this work: (a) preparation of RSV-niosomes, (b) encapsulation of RSV-niosomes in a biohydrogel made of κ-C and gelatin, (c) and *in vitro* release at 37 °C. Note: the location of RSV at the bilayer interface is speculated based on previous qualitative observations.

Briefly, the niosomes used in this studied were made upon equimolar mixture of two non-ionic surfactants (*i.e.*, Tw80 and Sp80). RSV was incorporated into the vesicles using the methodology previously described by some of us to encapsulate hydrophobic molecules in this type of niosomes (Fig. 1).^46^ The so obtained niosomes were subsequently mixed with a suitable amount of a mixture of κ-C and gelatin (1 : 1 mass ratio, 4% w/v) in water and gently heated. The hydrogel was obtained after cooling the mixture down to room temperature (RT) (ESI, Fig. S1†). The RSV-niosomes were incorporated into the hydrogel with a high encapsulation efficiency (EE) of 97.00 ± 0.02%. Moreover, the gelation ability of κ-C and gelatin were not significantly influenced by the presence of niosomes.

**Fig. 2:**
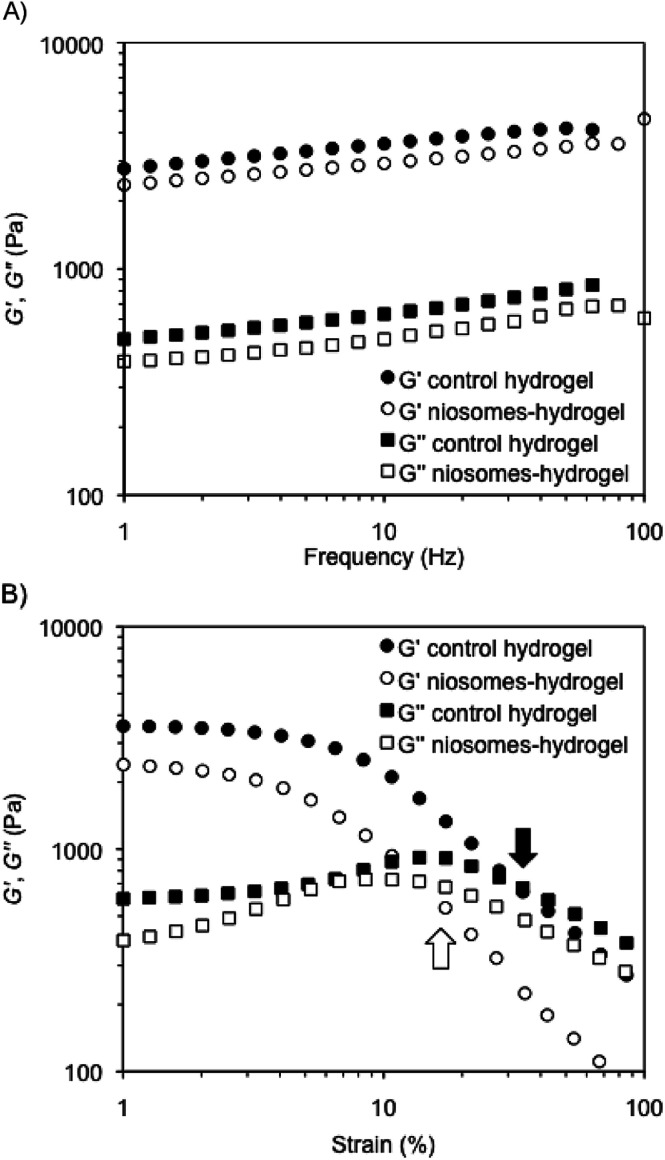
DFS and DSS measurements of (A) control hydrogel in the absence of niosomes (*i.e.*, κ-C : gelatin hydrogels (1 : 1 mass ratio, 4% w/v)), and (B) hydrogel containing RSV-niosomes. See Experimental section for details.

The mechanical properties of representative composite hydrogels were investigated by dynamic rheological measurements and compared to control gels prepared in the absence of niosomes. In general, the storage modulus (*G*′) and the loss modulus (*G*′′) were measured at 25 °C in water as a function of frequency (DFS, dynamic frequency sweep), strain (DSS, dynamic strain sweep), and time (DTS, dynamic time sweep) (see Experimental section). The viscoelastic nature of the samples was evidenced by *G*′ values of *ca.* one order of magnitude greater than *G*′′ within the linear regime, as well as a low dependence of *G*′ with the applied frequency (*i.e.*, *G*′ ≈ *ω*^0.09^ within the range 1–100 Hz). In agreement with our previous observations,^49^ the encapsulation of RSV-niosomes into κ-C : gelatin hydrogels (1 : 1 mass ratio, 4% w/v) did not change the mechanical properties of the network, which displayed similar moduli (*G*′ ≈ 3 × 10^3^ Pa) and nearly the same dissipation factor values (tan *δ* = *G*′′/*G*′ ≈ 0.15 ± 0.01) (Fig. 2A). These results indicated a good response of the hydrogels to external forces. DSS measurements confirmed that all hydrogels remained stable during the application of an oscillation stress within the linear viscoelastic regime (Fig. 2B). The results showed that the incorporation of the niosomes reduced significantly the resistance of the hydrogels against the shear stress (*γ*) until *G*′′ exceeded *G*′ (*i.e.*, critical *γ* at break (*γ*_c_) ≈ 32%) (control native hydrogel) *vs.* 14% (hydrogel containing RSV-niosomes). Similar destabilization was previously observed in gel networks made of κ-C and methylcellulose.^49^ Finally, DTS (dynamic time sweep) measurements confirmed the temporal stability of the hydrogels within the linear viscoelastic regime (1 Hz frequency, 0.1% strain) (ESI, Fig. S2†).

**Fig. 3:**
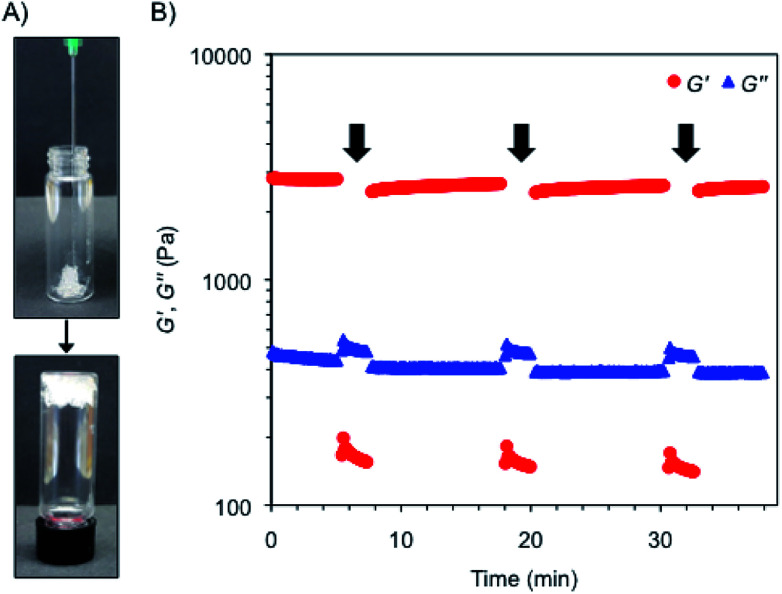
(A) Injectability property of the κ-C : gelatin hydrogel (1 : 1 mass ratio, 4% w/v) containing RSV-niosomes. (B) Rheological loop test of the previous composite hydrogel. Steps with high shear strain are indicated with a black arrow.

Interestingly, the bulk hydrogels were found to be injectable, a fundamental requisite for numerous biomedical applications.^47,48^ The injectability was confirmed by instantaneous re-gelation after flowing the hydrogel without clogging through a 21-gauge needle (Fig. 3A). In good agreement, a thixotropic behavior was demonstrated by a 3-step rheological test consisting of (1) application of a low shear strain (0.1%) at 1 Hz frequency for 5 min, as defined by DTS experiments (gel phase, *G*′ > *G*′′); (2) increase of the shear strain to *ca.* 50% to induce the collapse of the gel (liquid phase, *G*′′ > *G*′) and maintenance for 2 min; and (3) reduction at the same rate to the initial shear strain and maintenance for 10 min to stabilize the recovered network (gel phase, *G*′ > *G*′′). The loop was repeated three times achieving nearly full recovery (>95%) of the gel strength within a few minutes after each cycle (Fig. 3B).

### 3.2. Stability of RSV-niosomes after encapsulation and hydrogel degradation

**Fig. 4:**
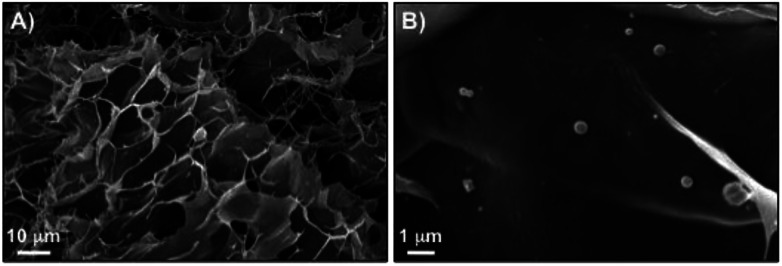
FE-SEM images of xerogels prepared by freeze-drying the κ-C : gelatin (1 : 1 mass ratio, 4% w/v) hydrogel containing RSV-niosomes.

We have previously confirmed the stability and morphology preservation of nioplexes encapsulated in κ-C hydrogels (4% w/v). The average diameter of the vesicles slightly increases (<10 nm) after gel network degradation.^49^ Similarly, the structural integrity of niosomes entrapped in the hydrogel made of κ-C : gelatin (1 : 1 mass ratio, 4% w/v) was confirmed using dynamic light scattering (DLS) and emission field scanning electron microscopy (FE-SEM). Hydrogel degradation was induced by diluting the hydrogel to achieve a gelator concentration below the critical gelation concentration (*i.e.*, <2% w/v in the mixture κ-C : gelatin 1 : 1 mass ratio) and incubating the sample 24 h at 37 °C to trigger erosion and liberate the corresponding niosomes into the receptor phase. DLS measurements of non-encapsulated RSV-niosomes showed a mean diameter of 90.2 ± 0.4 nm with a PDI value of 0.15 ± 0.01 (ESI, Fig. S6†). The average diameter remained similar (96.1 ± 0.9 nm) with homogeneous size distribution (0.434 ± 0.007 nm) after encapsulation of the niosomes in the hydrogel and subsequent release. These findings were also corroborated by FE-SEM, which showed the presence of spherical niosomes^46^ within the κ-C : gelatin polymer matrix with a size distribution in agreement with DLS data (Fig. 4). The observed increase in PDI after hydrogel degradation is in agreement with our previous observations^49^ and could be attributed to non-specific intermolecular interactions between the functional groups of the biopolymers and the niosomes affecting to some extent the supramolecular organization of the dynamic vesicles.

### 3.3. *In vitro* release studies

*In vitro* release studies of the hydrogels containing RSV-niosomes were performed at 37 °C and at pH 1.2 and 6.8 to emulate gastrointestinal conditions. As a reservoir for the release, hydrogels were immersed in buffer solution at the corresponding pH and aliquots were removed at regular intervals. RSV was analyzed by UV-vis spectroscopy according to a proper standard curve in water (*r*^2^ = 0.987) (ESI, Fig. S7–S8†). The removed volume was always replaced by the same amount of fresh buffer.

**Fig. 5:**
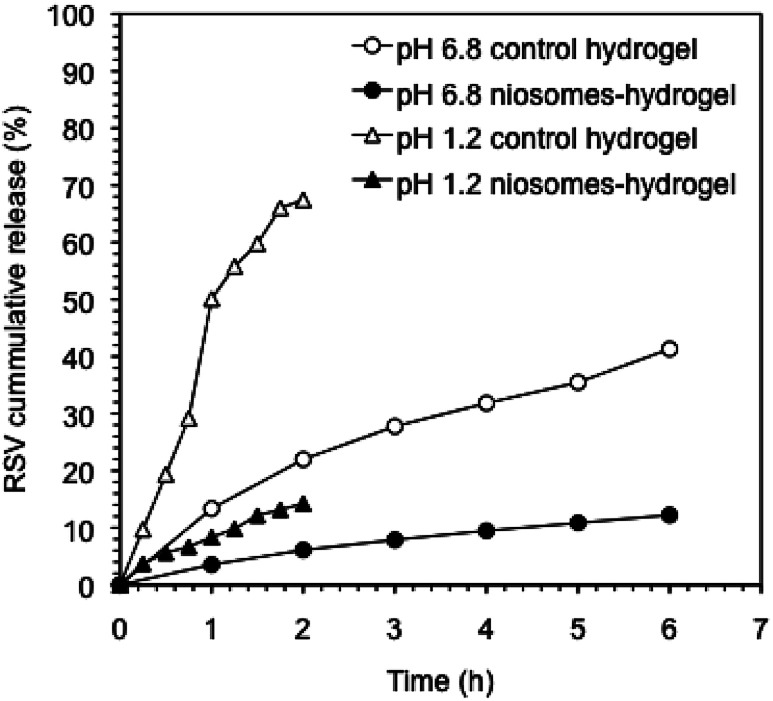
Cumulative release of RSV from control hydrogels and RSV-niosomes encapsulated in hydrogels at pH 1.2 and 6.8. The hydrogels consisted of a mixture κ-C : gelatin (1 : 1 mass ratio, 4% w/v).

The release profile of RSV from the hydrogel containing RSV-niosomes was compared with that obtained from the hydrogel bearing RSV without niosomes (Fig. 5). The results showed that 67% of RSV was released from the control hydrogel under gastric conditions (pH 1.2) after 2 h, whereas 14% was released from the hydrogel containing RSV-niosomes within the same period of time. Under intestinal conditions (pH 6.8), 41% and 25% of RSV was released from control hydrogel and hydrogel containing RSV-niosomes after 6 h, respectively. These values were considerably lower than those obtained for the RSV release from the niosomes alone (without hydrogel), using a dialysis membrane (86% at pH = 1.2, 2 h, and 97% at pH = 6.8, 6 h). In this sense, the RSV release was significantly delayed by the inclusion of the niosomes in the hydrogel. The use of niosomes enables to slow down the release of RSV from the hydrogel matrix preventing an initial burst release. A similar behavior has been observed in the release of 5-carboxyfluorescein from liposomes encapsulated in chitosan-β-glycerophosphate hydrogel, since 100% of the probe was released from the hydrogel without liposomes in 2 h, while 20% was release from the proposed formulation in 14 h.^50^ Moreover, the release behavior at different pH values could also be correlated, at least to some extent, with the mechanical stability of the network with the shear stress (*γ*). Although the dissipation factor of the gels remained essentially constant when the pH was reduced to 1.2, the *γ* at break was reduced one order of magnitude compared to that at neutral pH (*i.e.*, *γ*_c_ ≈ 8.6% (control hydrogel) and 4.6% (niosome-hydrogel)) (Fig. S3–S5†). Thus, charge neutralization of the ionic gel at low pH critically destabilizes the network, which facilitates the subsequent release of the niosomes.

**Fig. 6:**
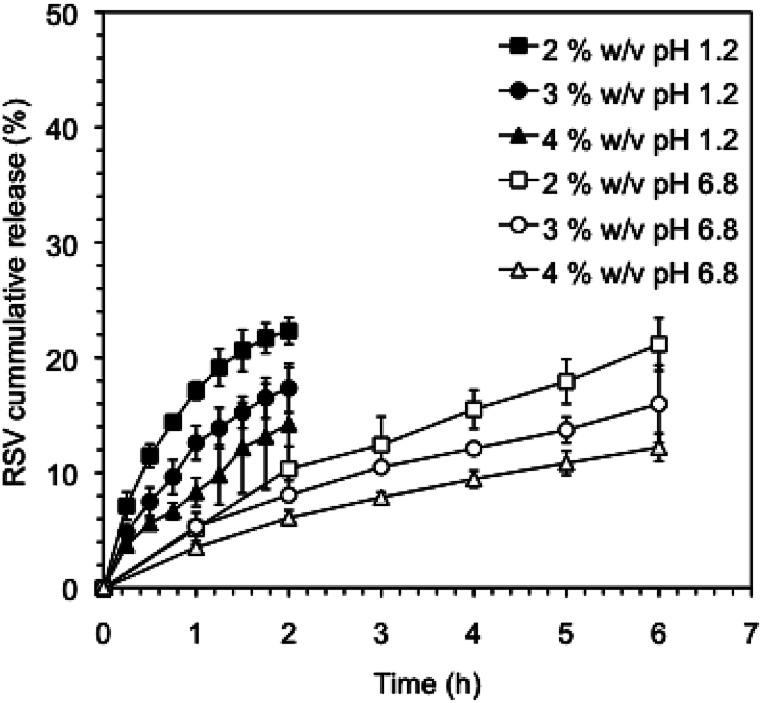
Cumulative release of RSV from RSV-niosomes encapsulated in κ-C : gelatin (1 : 1 mass ratio) hydrogels using different concentrations of the gelator system (*i.e.*, 2%, 3% and 4% w/v) at pH 1.2 and 6.8.

It is well known that the rate of drug release can be modulated by crosslinking of the gel networks and/or modifying the network density using different gelator concentrations.^51^ In this way, we could tune the RSV release by decreasing the κ-C : gelatin total concentration. Fig. 6 shows the cumulative release profiles of RVS-niosomes from these hydrogels at different gelators concentrations. Specifically, the RSV release percentage observed at pH 1.2, after 2 h, enhanced *ca.* 1.5 times when the total gelator concentration was reduced to half (*i.e.*, 14% and 22% release at a gelator system concentration of 4% w/v and 2% w/v, respectively). Similarly, the percent of released RSV at pH 6.8, after 6 h, increased *ca.* 1.75 times by reducing the gelator concentration to half (*i.e.*, 12% at 4% w/v and 21% at 2% w/v, respectively). This is in agreement with a faster diffusion release through a less dense network.

The observed behavior is similar to that reported by Cao and co-workers, who developed a supramolecular hydrogel based on tetraethylammonium 3-{[(2*R*)-2-(octadecylamino)-3-phenyl propanoyl]amino}butyrate, TC_18_PheBu, for salicylic acid release.^52^ Specifically, the drug release enhanced from 48.6% to 58.8% after 10 h when the hydrogelator concentration was reduced from 8% w/w to 4% w/w. Moreover, we recently also reported the efficient encapsulation of nucleic acids in hybrid hydrogels made of *N*-protected phenylalanine (Fmoc-Phe-OH) and κ-C.^53^ The nucleic acid release was 80% and 100% after 5 h when the κ-C concentration was adjusted to 1% and 0.8%, respectively. Besides, the presence of κ-C in the formulation avoided the initial burst effect, probably due to the increase of the entanglement density of the non-covalently crosslinked hydrogels.^53^

### 3.4. Drug release mathematical models

The release data obtained for all systems were fitted according to four theoretical models that describe the diffusion-related drug release from polymeric matrices (see Experimental section). The selected models are defined by the first order^54^ equation [eqn (3),], Higuchi^55^ equation [eqn (4)], Korsmeyer–Peppas^56^ equation [eqn (5)], and Weibull^57,58^ equation for delayed release [eqn (6)]. The first order, Higuchi, and Korsmeyer–Peppas models are short time approximations, being generally limited to the first 60% of the release profile.

The total RSV cumulative release from all hydrogel matrices at pH 1.2 was found to follow the first order kinetic model (*i.e.*, their regression coefficients were greater (*r*^2^ > 0.99) than those obtained with the other theoretical models^59^ (ESI, Tables S1–S7, and Fig. S9–S32†)). According to Peppas' equation, the RSV release afforded *n* (diffusion coefficient) values in this condition ranging from 0.50 to 0.70. The same result was also found when the gelator system concentration was adjusted to 2% w/v at pH 6.8 (*n* = 0.72). This indicates that the release process is governed by anomalous diffusion (non-Fickian) (0.5 < *n* < 1). Using a gelator system concentration of 4% w/v at pH 6.8, the first order and Korsmeyer–Peppas models were in good agreement with regression coefficients greater than 0.99 and *n* values of 0.65. However, at a concentration of 3% w/v both Peppas and Weibull's models showed good fitting with an *n* value of 0.61. All these exponent values are in close agreement with those predicted for non-Fickian diffusions. This anomalous transport suggests the presence of specific interactions and/or erosion mechanisms between niosomes and the fibrous gel matrices. Overall, these results suggest a diffusion-controlled release as the main process that govern the liberation of RSV from the κ-C : gelatin hydrogel matrices.

### 3.5. Stability of encapsulated *trans*-RSV against UV light

**Fig. 7:**
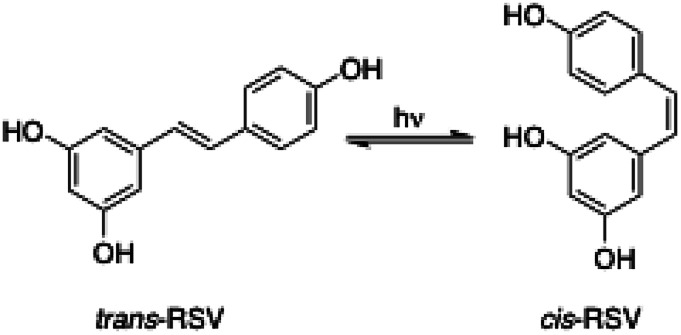
*trans*/*cis* Photoisomerization of RSV induced by light irradiation.

The reversible photoinduced *trans*–*cis* isomerization of stilbenes has been extensively studied in the last decades.^60^ In the case of stilbenoid RSV, the *trans*-isomer is the most stable from the steric point of view^61^ and the active biologically compound.^62,63^ However, the *cis*/*trans* equilibrium position (Fig. 7) is significantly influenced not only by light irradiation but also by the RSV concentration, pH and temperature.^64^ For instance, standard laboratory lighting conditions favor an equilibrium of about 91% *cis*-RSV.^61^ Thus, the protection of RSV against photoisomerization is important in the design of new delivery systems.

In this work, RSV ethanolic solution, RSV-niosome solution and RSV-niosome entrapped hydrogel were evaluated with respect to *trans*-to-*cis* photoisomerization under UV irradiation (*λ* = 365 nm) during 1 h. All the samples presented the same initial *trans*-RSV concentration and the quantification of the isomerization equilibrium was performed by HPLC according to the corresponding calibration curve. The results were expressed as molar ratio of *trans*-RSV after and before UV light irradiation (ESI, Fig. S33–S34†). The molar ratio obtained for the control ethanolic solution was 0.26, whereas the values obtained for niosomes and niosomes-hydrogel were 0.70 and 0.89, respectively (ESI, Table S8†). These results suggest a significant protective effect against photoisomerization due to the encapsulation of RSV within niosomes in comparison with unencapsulated RSV in solution. This effect even slightly enhanced upon encapsulation of the niosomes in the hydrogel matrix. It is worth mentioning that this kind of studies has scarcely been performed using hydrogels or vesicles as delivery systems individually, and no previous works were found in hybrid systems such as niosomes-hydrogels. However, the protective effect provided by others RSV delivery systems such as particles or emulsions has been previously studied. For instance, Koga and co-workers used sodium caseinate microparticles for RSV delivery and observed photoisomerization protection (under 365 nm irradiation during 1 h) with a *trans*/*cis* molar ratio of 0.65, while unencapsulated RSV presented a molar ratio of 0.49.^65^ Additionally, Liu and co-workers characterized protein–polyphenolic conjugate nanoparticles loaded with curcumin and RSV.^66^ After UV light irradiation for 1 h, the retention of *trans*-RSV dissolved in DMSO was 65%, while the retention of *trans*-RSV entrapped in nanoparticles was 90%. This effect was attributed to the presence of the nanoparticles, which decrease the amount of UV light reaching the *trans*-RSV due to light scattering or absorption effects.^66^ More recently, Kumar and co-workers also observed photoprotection of *trans*-RSV against isomerization when it was encapsulated in nanoemulsions such as inclusion complexes withβ-cyclodextrin (*i.e.*, isomerization of *trans*-RSV was 64% and 25%, in a mixture water/ethanol and in a nanoemulsion, respectively). Therefore, the photoprotection of *trans*-RSV provided by the niosome-hydrogel system described in this work is similar or better than some of the previous reported systems.

## 4. Conclusions

The incorporation of RSV-niosomes in biohydrogels made of κ-C and gelatin provides a versatile delivery system for RSV. The formulation presents good mechanical properties such as thixotropy and rigidity. In addition, the structural integrity of the niosomes is maintained after encapsulation in the hydrogels. It is also possible to modulate the release of the encapsulated RSV by modifying the total concentration of biopolymers in the hydrogel. Furthermore, the encapsulation of RSV in the niosome-hydrogel systems protects it from the photoisomerization, maintaining its biologically active *trans*-isomer in higher concentration for subsequent administration. Further biological studies of this platform for the delivery of RSV in food and pharmaceutical applications are currently underway in our laboratories.

## Conflicts of interest

There are no conflicts of interest to declare.

## Supplementary Material

RA-009-C8RA09655D-s001
