# Supplementary material for: Niosomes encapsulated in biohydrogels for tunable delivery of phytoalexin resveratrol
Source: RSC Adv. 2019 Mar 8;9(14):7601–9. doi: 10.1039/c8ra09655d (PMC9061210; doi:10.1039/c8ra09655d)
Supplement: RA-009-C8RA09655D-s001 [file RA-009-C8RA09655D-s001.pdf]

Electronic Supplementary Material (ESI) for RSC Advances.
This journal is © The Royal Society of Chemistry 2019



# *Supporting Information*

## Niosomes encapsulated in biohydrogels for tunable delivery of phytoalexin resveratrol

Noelia D. Machado,[a,b] Mariana A. Fernández,[a] Marleen Haering,[b] César Saldías[c] and David Díaz Díaz[b,d]*

[a]Instituto de Investigaciones en Físico-Química de Córdoba (INFIQC-CONICET), Departamento de Química Orgánica, Facultad de Ciencias Químicas, Universidad Nacional de Córdoba, Ciudad Universitaria, X5000HUA, Córdoba (Argentina). [b]Institute of Organic Chemistry, University of Regensburg, Universitätstrasse. 31, 93040 Regensburg (Germany). [c]Departamento de Química Física, Facultad de Química, Pontificia Universidad Católica de Chile, Macul, Santiago (Chile). [d]Instituto de Productos Naturales y Agrobiología del CSIC, Avda. Astrofísico Francisco Sánchez 3, 38206 La Laguna, Tenerife (Spain).

E-mail: David.Diaz@chemie.uni-regensburg.de; d.diaz.diaz@ipna.csic.es

**LIST OF CONTENTS**

*Page*

1. Digital pictures of representative hydrogels ………………………………… S3

2. Additional rheological plots ……………….............................................. S4

3. Stability of the niosomes in the hydrogels…………………………………... S6

4. Standard curves for RSV ..………………..……………………………….... S7

5. Model release parameters and fitting curves ………………………………... S8

6. Photoisomerization experiments ……………………………………………. S34

## 1. Digital pictures of representative hydrogels

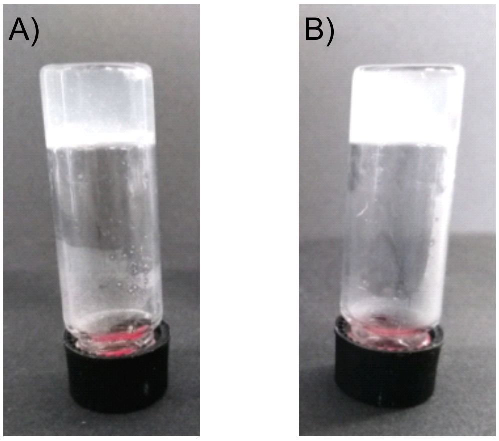


**Figure S1.** Representative hydrogel pictures: A) κ-C:gelatin (1:1 mass ratio, 4 % w/v). B) κ-C:gelatin (1:1 mass ratio, 4 % w/v) containing RSV-niosomes.

## 2. Additional rheological plots

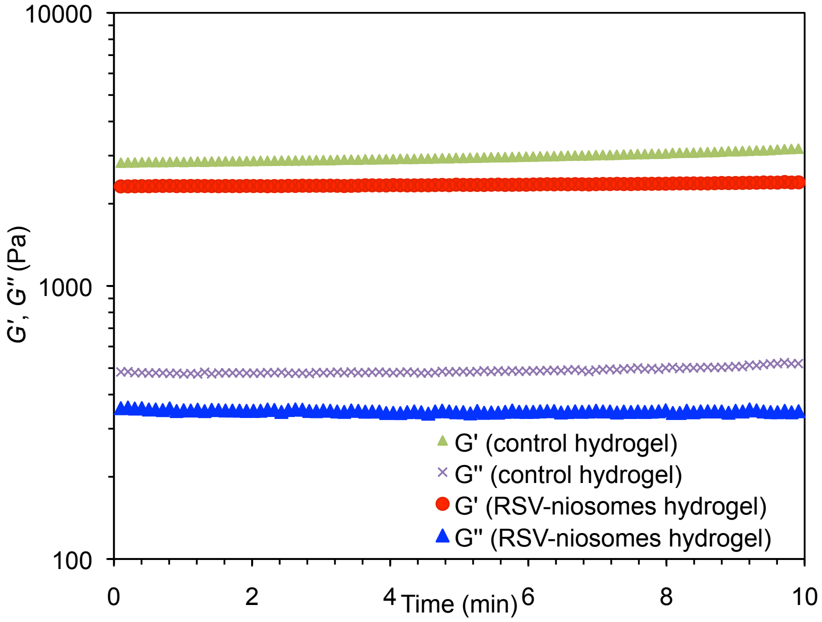


**Figure S2.** DTS measurements of native hydrogel (control hydrogel without niosomes) and hydrogel containing RSV-niosomes.

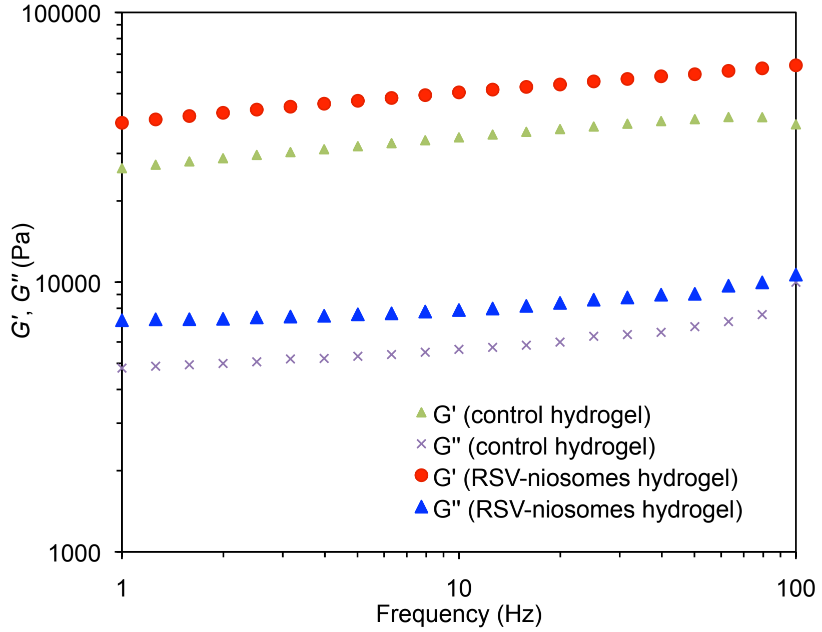


**Figure S3.** DFS measurements at pH 1.2 of native hydrogel (control hydrogel without niosomes) and hydrogel containing RSV-niosomes.

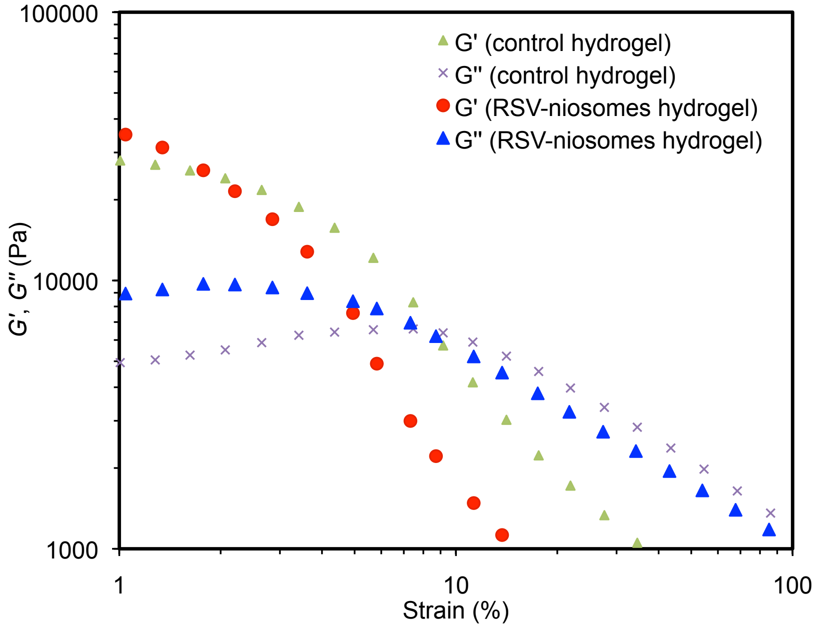


**Figure S4.** DSS measurements at pH 1.2 of native hydrogel (control hydrogel without niosomes) and hydrogel containing RSV-niosomes.

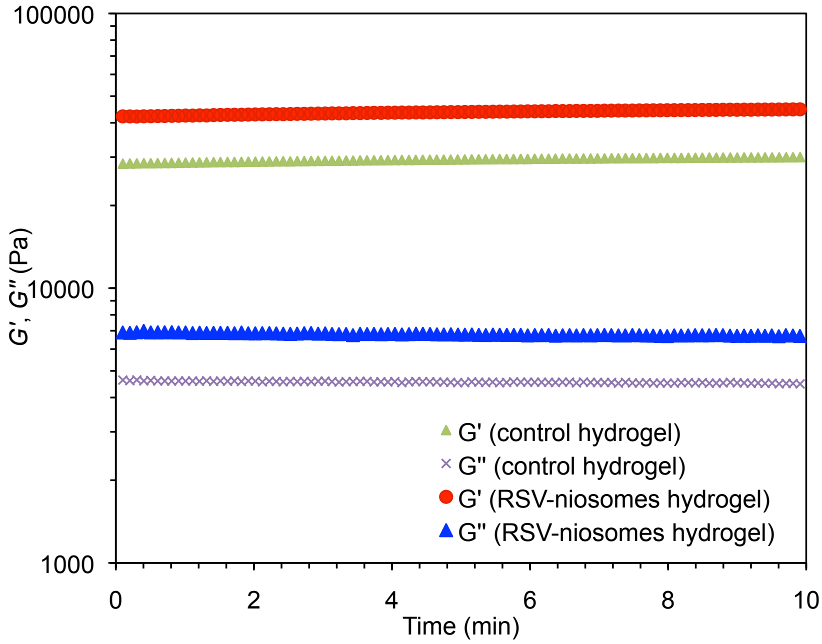


**Figure S5.** DTS measurements at pH 1.2 of native hydrogel (control hydrogel without niosomes) and hydrogel containing RSV-niosomes.

## 3. Stability of the niosomes in the hydrogels

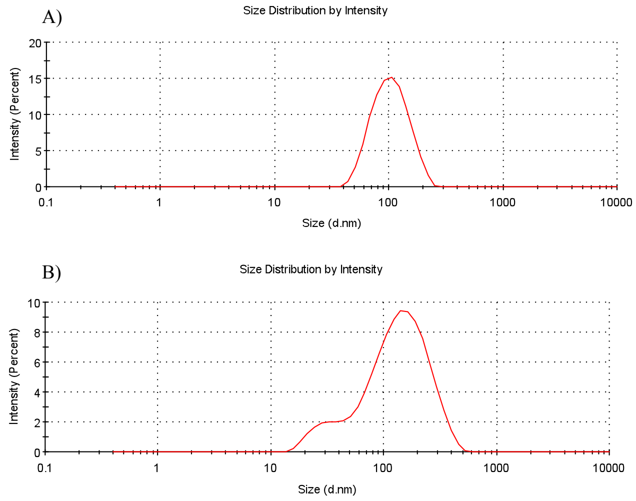


| Graph | Mean diamater (nm) | PDI |
|---|---|---|
| A | 90.2 ± 0.4 | 0.15 ± 0.01 |
| B | 96.1 ± 0.9 | 0.434 ± 0.007 |

**Figure S6.** Mean diameter of RSV-niosomes A) incubated at 37 ºC for 24 h and B) after hydrogel degradation.

## 4. Standard curves for RSV

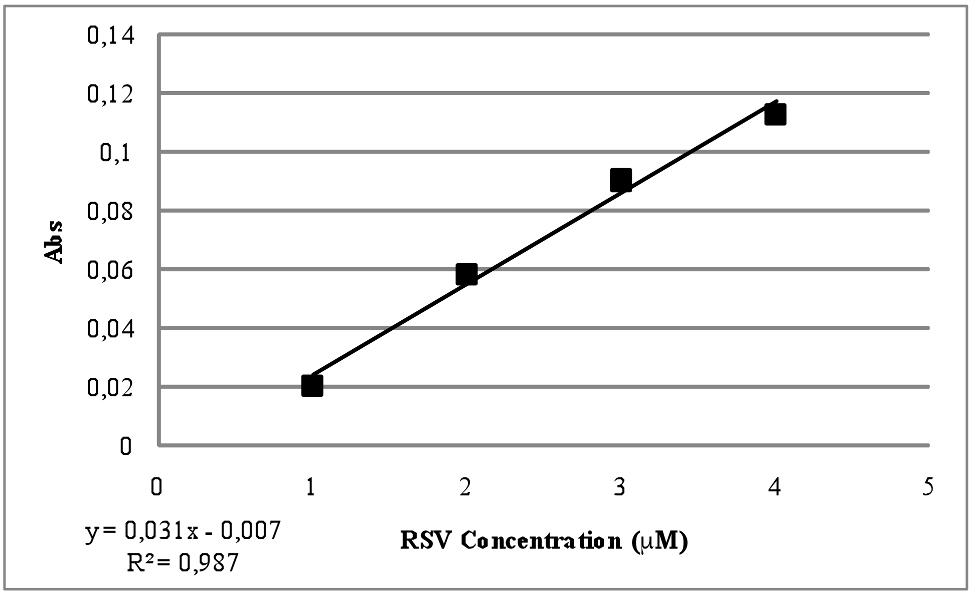


**Figure S7.** Standard curve of RSV in water using UV-visible spectrophotometry ($\lambda_{máx}$= 310 nm, cuvette path length 1 cm).

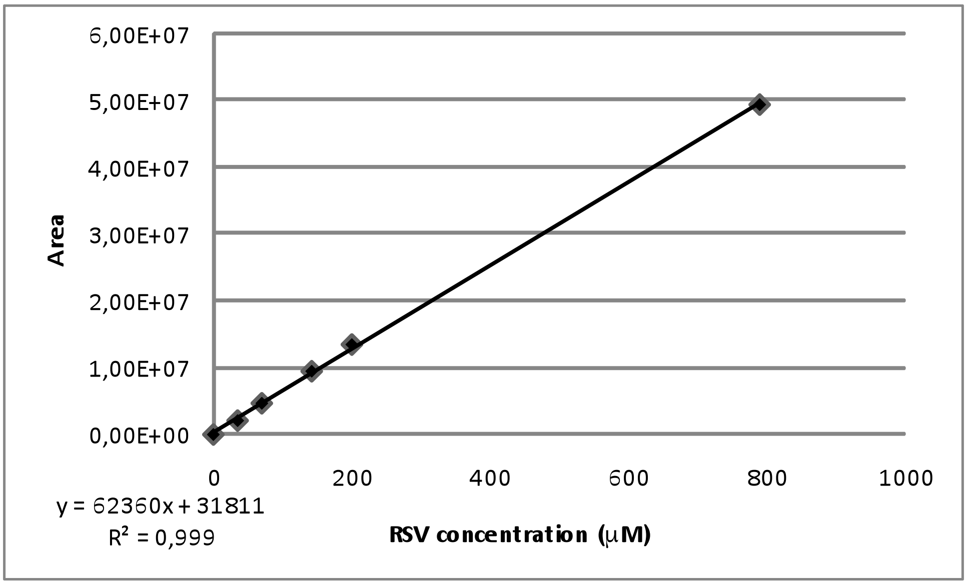


**Figure S8.** Standard curve of RSV using HPLC.

## 5. Model release parameters

**Table S1.** Model release parameters for *hydrogels* **κ-C:gelatin 4 % w/v** at pH 1.2, according to different mathematical models.

| Mathematical model | *k* | $r^2$ |
|---|---|---|
| First order | -0.0535 | 0.992 |
| Higuchi | 0.21 | 0.968 |
| Korsmeyer-Peppas | 2.85 (n= 0.7) | 0.986 |
| Weibull | -2.94× $10^{24}$ (a= 0.002, b= 0.05) | 0.985 |

**Table S2.** Model release parameters for *hydrogels* **κ-C:gelatin 3 % w/v** at pH 1.2 according to different mathematical models.

| Mathematical model | *k* | $r^2$ |
|---|---|---|
| First order | -0.7189 | 0.997 |
| Higuchi | 0.28 | 0.989 |
| Korsmeyer-Peppas | 3.22 (n= 0.60) | 0.993 |
| Weibull | -2.94× $10^{24}$ (a= 0.005, b= 0.05) | 0.994 |

**Table S3.** Model release parameters for *hydrogels* **κ-C:gelatin 2 % w/v** at pH 1.2 according to different mathematical models.

| Mathematical model | *k* | $r^2$ |
|---|---|---|
| First order | -1.070 | 0.998 |
| Higuchi | 0.38 | 0.992 |
| Korsmeyer-Peppas | 3.69 (n= 0.50) | 0.985 |
| Weibull | -2.94× $10^{24}$ (a= 0.013, b= 0.04) | 0.988 |

**Table S4.** Model release parameters for *hydrogels* **κ-C:gelatin 4 % w/v** at pH 6.8, according to different mathematical models.

| Mathematical model | *k* | $r^2$ |
|---|---|---|
| First order | -0.1366 | 0.998 |
| Higuchi | 0.11 | 0.984 |
| Korsmeyer-Peppas | 1.06 (n= 0.65) | 0.998 |
| Weibull | -2.94× $10^{24}$ (a= -0.002, b= 0.05) | 0.997 |

**Table S5.** Model release parameters for *hydrogels* **KC:gelatin 3 % w/v** at pH 6.8 according to different mathematical models.

| Mathematical model | *k* | $r^2$ |
|---|---|---|
| First order | -0.1062 | 0.991 |
| Higuchi | 0.14 | 0.991 |
| Korsmeyer-Peppas | 2.14 (n= 0.61) | 0.998 |
| Weibull | -2.94× $10^{24}$ (a= -0.019, b= 0.05) | 0.998 |

**Table S6.** Model release parameters for *hydrogels* **κ-C:gelatin 2 % w/v** at pH 6.8 according to different mathematical models.

| Mathematical model | *k* | $r^2$ |
|---|---|---|
| First order | -0.1110 | 0.990 |
| Higuchi | 0.18 | 0.970 |
| Korsmeyer-Peppas | 2.21 (n= 0.72) | 0.986 |
| Weibull | -2.94× $10^{24}$ (a= 0.001, b= 0.05) | 0.988 |

**Table S7.** Correlation coefficients obtained by fitting the experimental data with First Order, Higuchi, Korsmeyer-Peppas and Weibull models.

| pH | Gel formulation κ-C:gelatin (1:1 mass ratio) w/v | Drug release mathematical models | | | |
|---|---|---|---|---|---|
| | | First order | Higuchi | Korsmeyer-Peppas | Weibull |
| 1.2 | 2 % | **0.998** | 0.992 | 0.985 | 0.988 |
| | 3 % | **0.997** | 0.989 | 0.993 | 0.994 |
| | 4 % | **0.992** | 0.968 | 0.986 | 0.985 |
| 6.8 | 2 % | **0.990** | 0.970 | 0.986 | 0.988 |
| | 3 % | 0.991 | 0.911 | **0.998** | **0.998** |
| | 4 % | **0.998** | 0.984 | **0.998** | 0.997 |

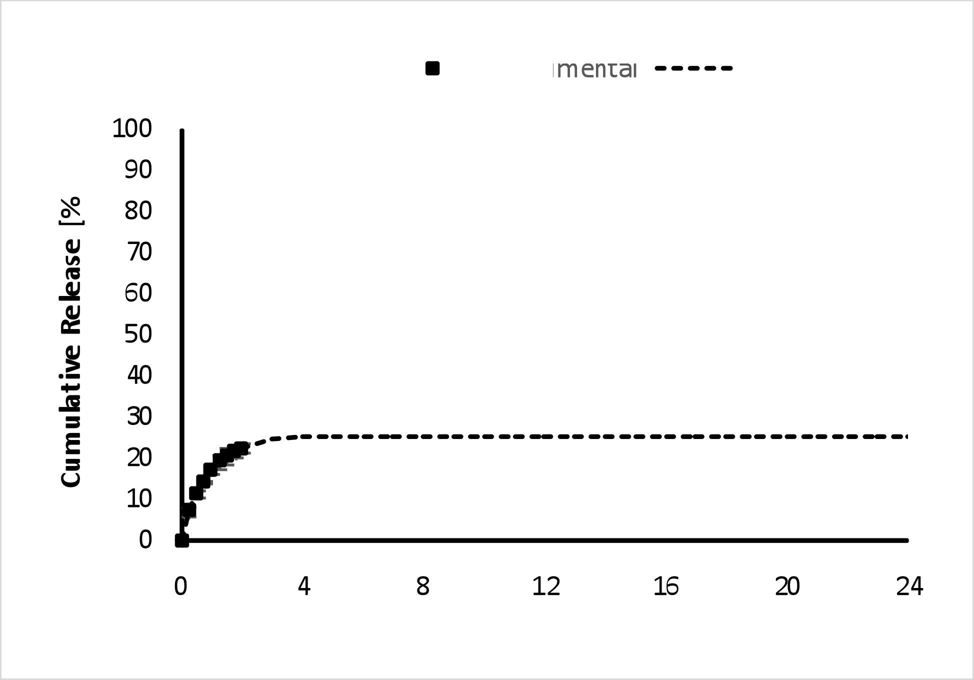


| y = y0 + A * exp(R0 * x) | y0 | A | R0 |
|---|---|---|---|
| | 25,27300464 | -23,721708 | -1,06978171 |

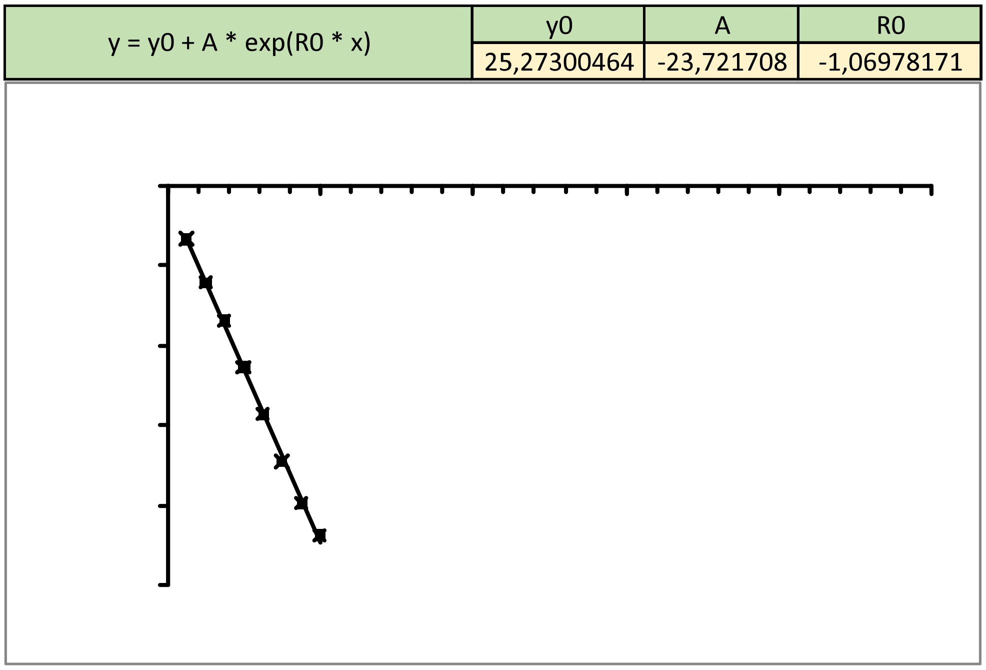

**Figure S9.** 2 % w/v κ-C:gelatin (1:1 mass ratio) hydrogels. First order kinetic model at pH 1.2.

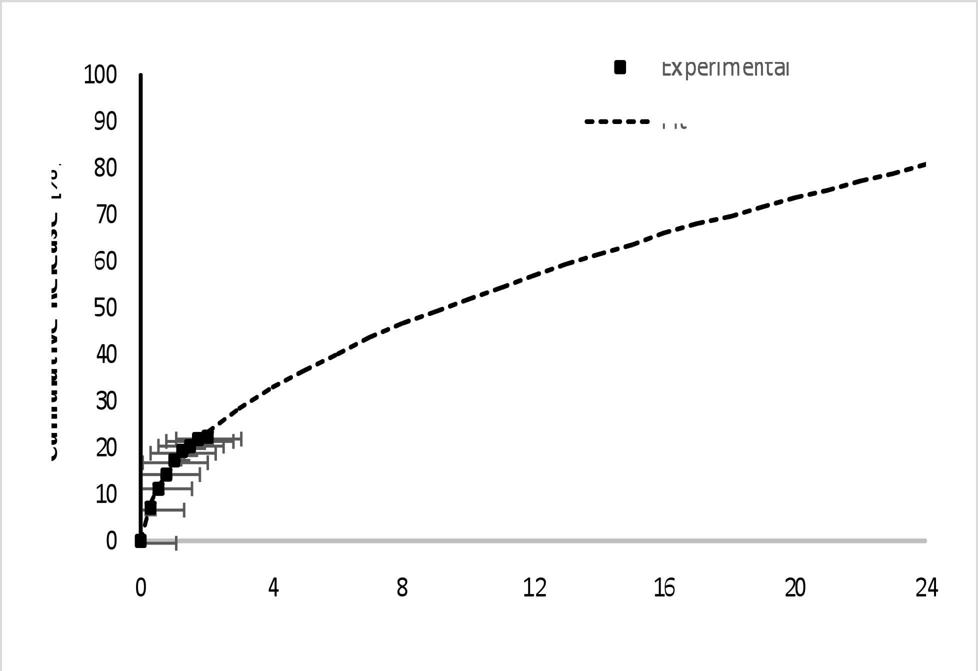


| $y = y_\infty * k * \sqrt{t}$ | $y_\infty$ | k |
|---|---|---|
| | 43,0351502 | 0,38268156 |

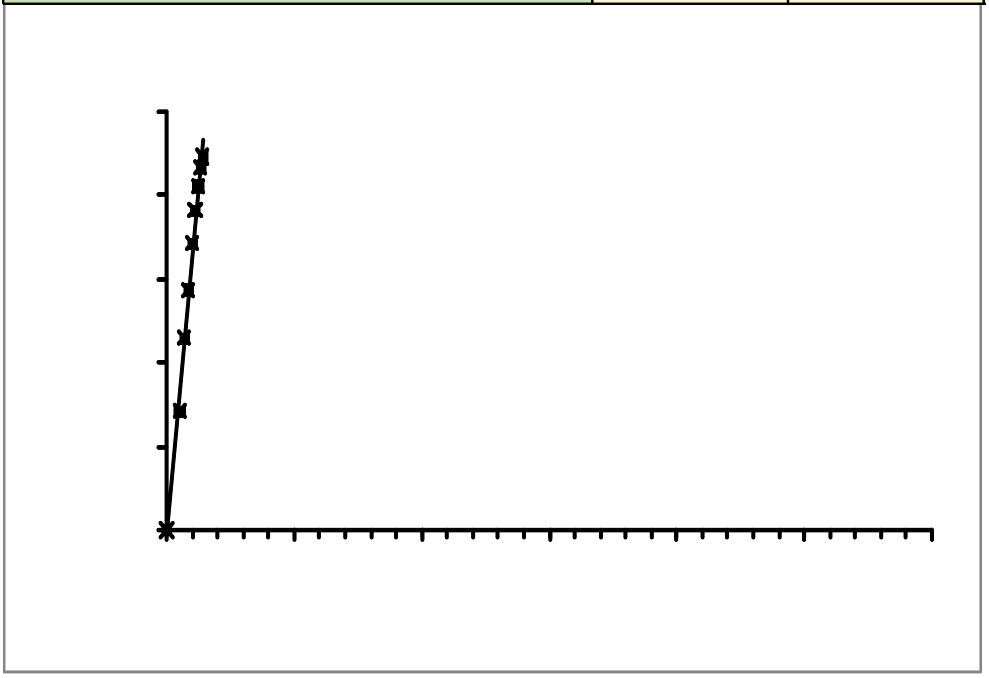

**Figure S10.** 2 % w/v κ-C:gelatin (1:1 mass ratio) hydrogels. Higuchi model at pH 1.2.

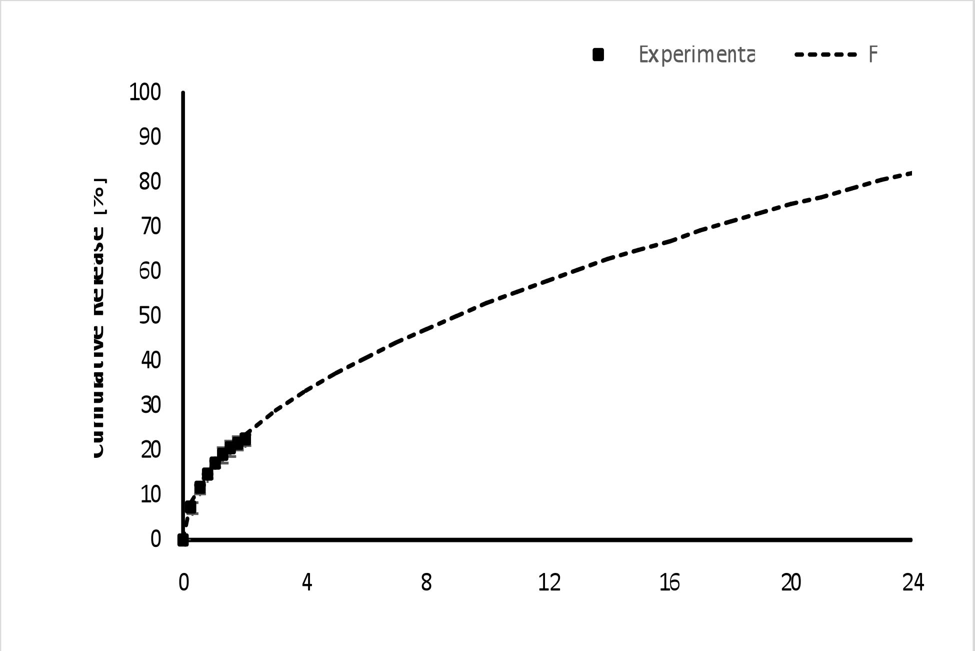


| $y = y\infty * k * t^n$ | y∞ | k | n |
|---|---|---|---|
| | 4,451031501 | 3,694046376 | 0,506153561 |

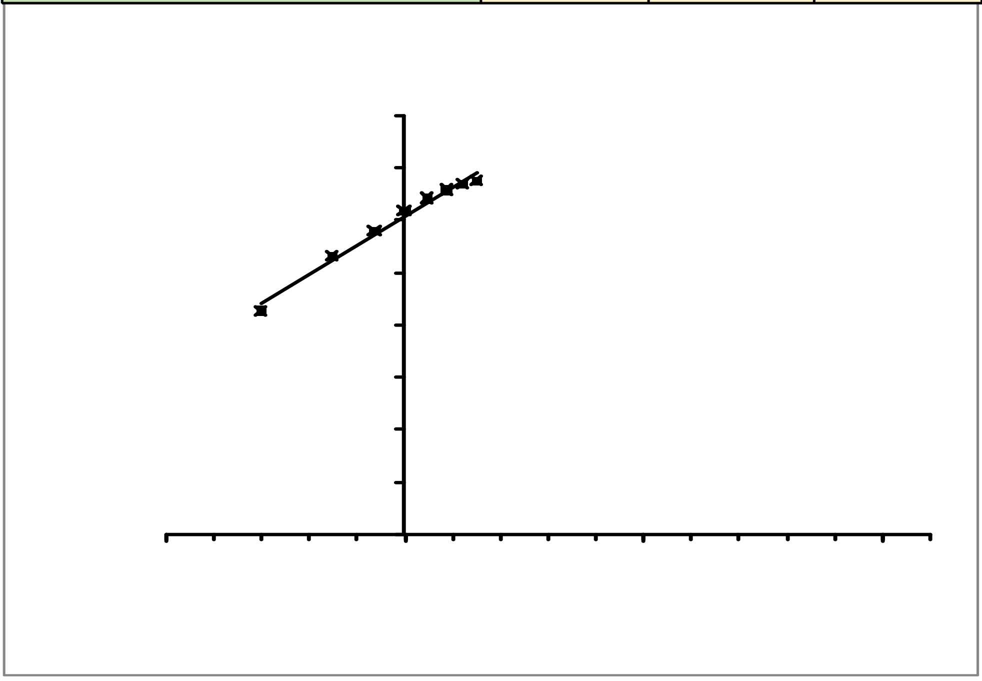

**Figure S11.** 2 % w/v κ-C:gelatin (1:1 mass ratio) hydrogels. Korsmeyer-Peppas model at pH 1.2.

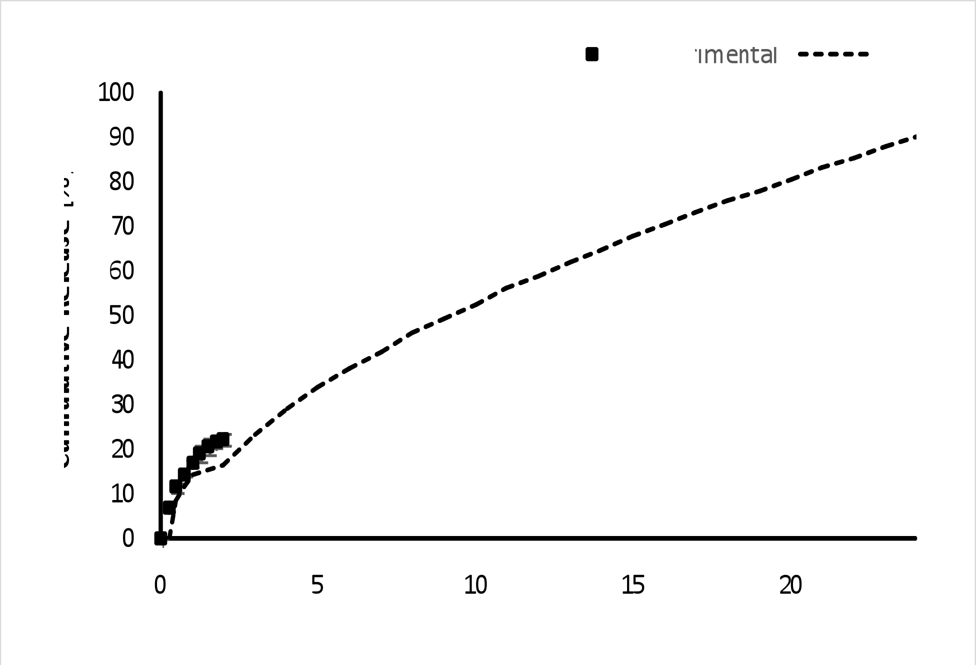


| $y = y_\infty * a * (1-\exp(-(kt)^b)$ | y∞ | a | k | b |
|---|---|---|---|---|
| | -0,01126133 | 0,013 | -2,94647E+24 | 0,04 |

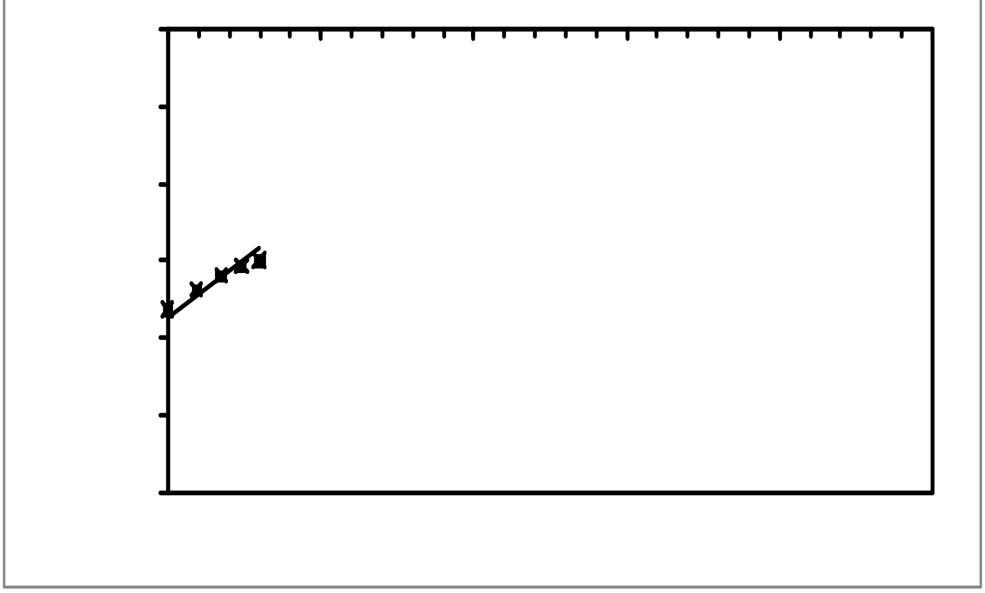

**Figure S12.** 2 % w/v κ-C:gelatin (1:1 mass ratio) hydrogels. Weibull model at pH 1.2.

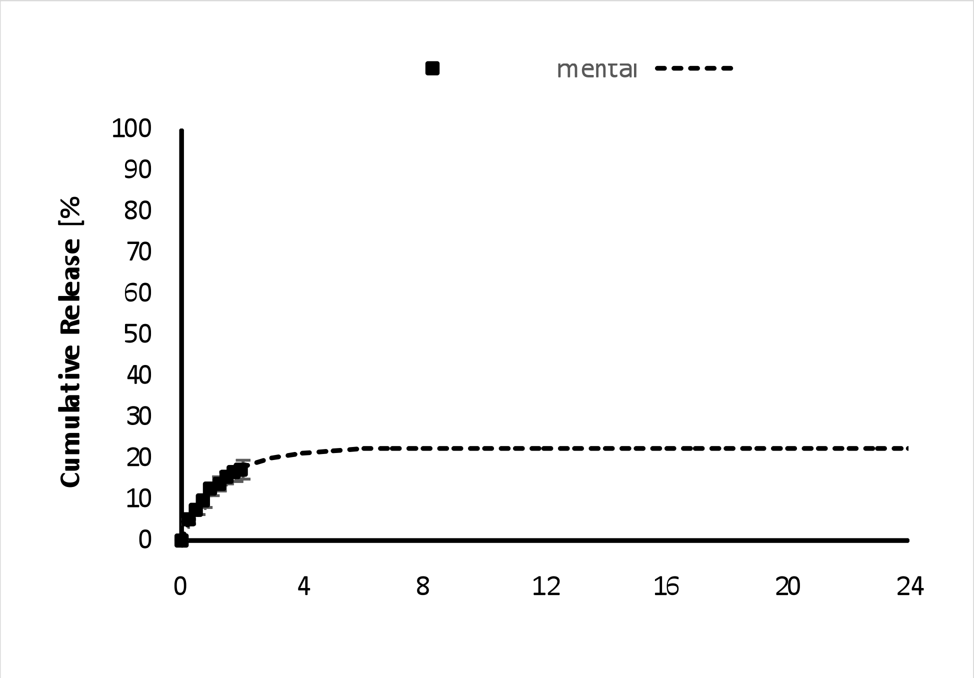


| y = y0 + A * exp(R0 * x) | y0 | A | R0 |
|---|---|---|---|
| | 22,52075146 | -21,323002 | -0,718924601 |

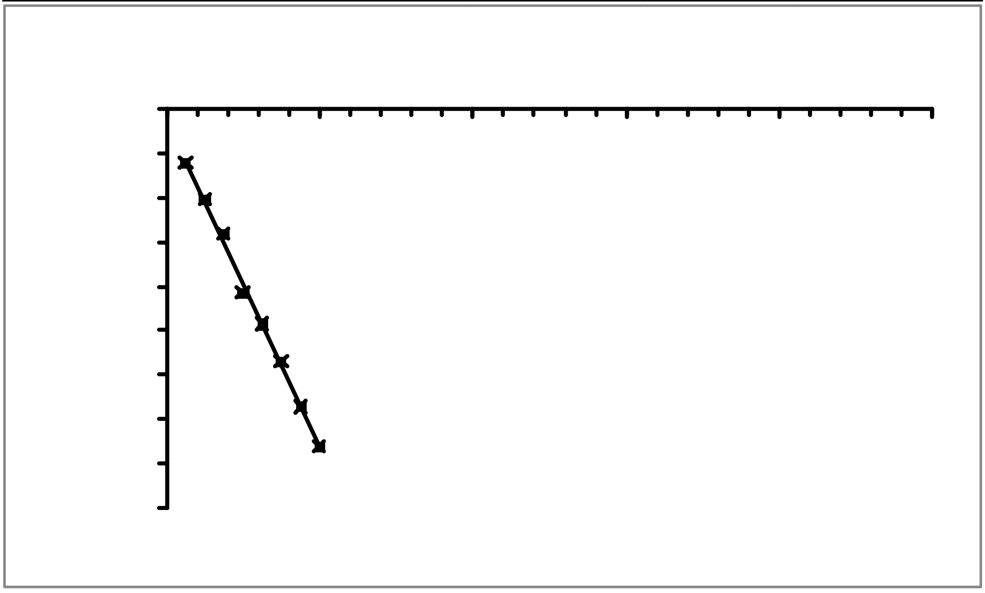

**Figure S13.** 3 % w/v κ-C:gelatin (1:1 mass ratio) hydrogels . First order kinetic model at pH 1.2.

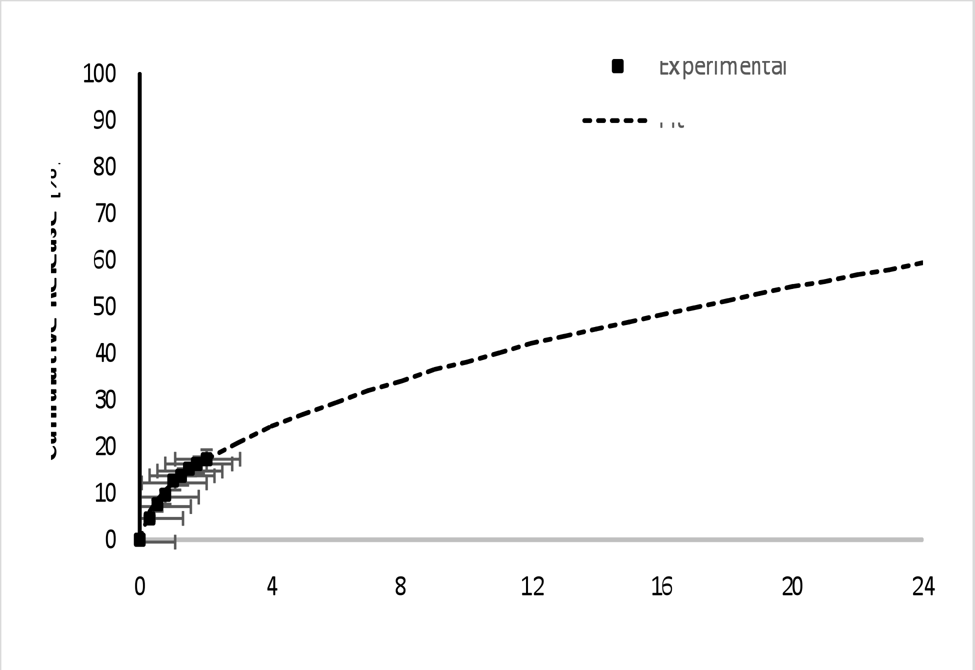


| y = y∞ * k * √t | y∞ | k |
|---|---|---|
| | 43,0341866 | 0,2820662 |

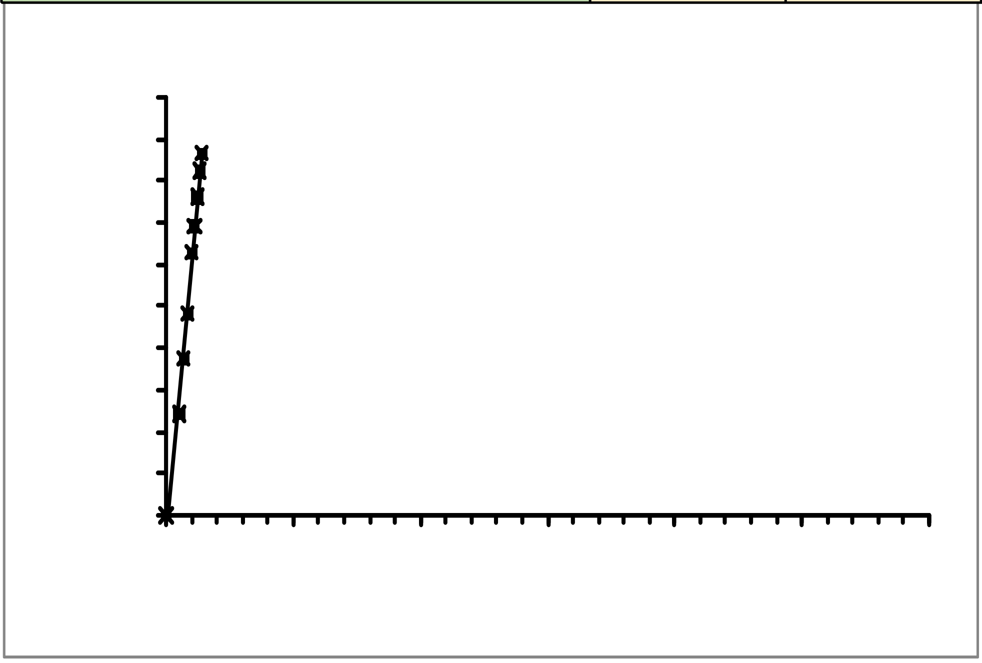

**Figure S14.** 3 % w/v κ-C:gelatin (1:1 mass ratio) hydrogels . Higuchi model at pH 1.2.

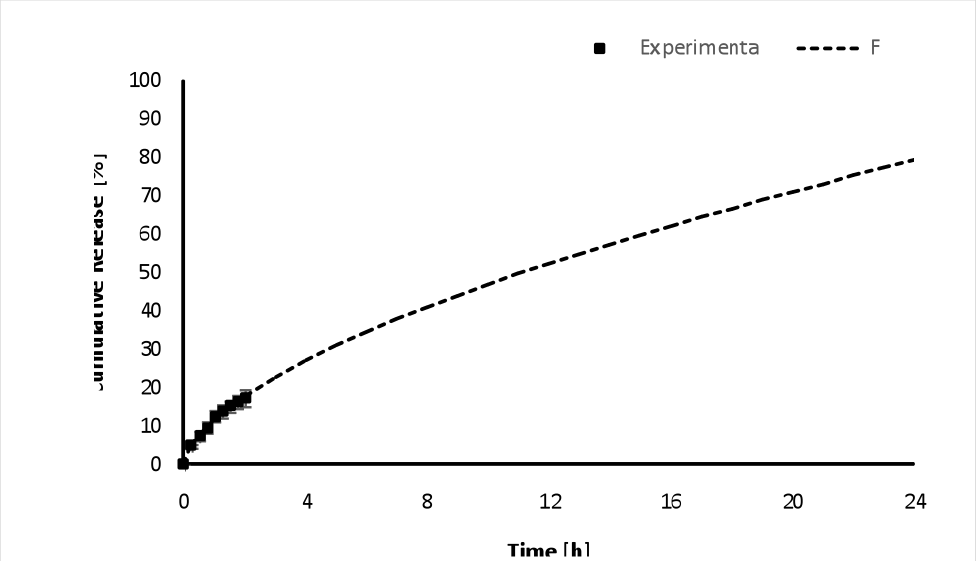


| $y = y_\infty * k * t^n$ | $y_\infty$ | k | n |
|---|---|---|---|
| | 3,666458912 | 3,22361686 | 0,598440286 |

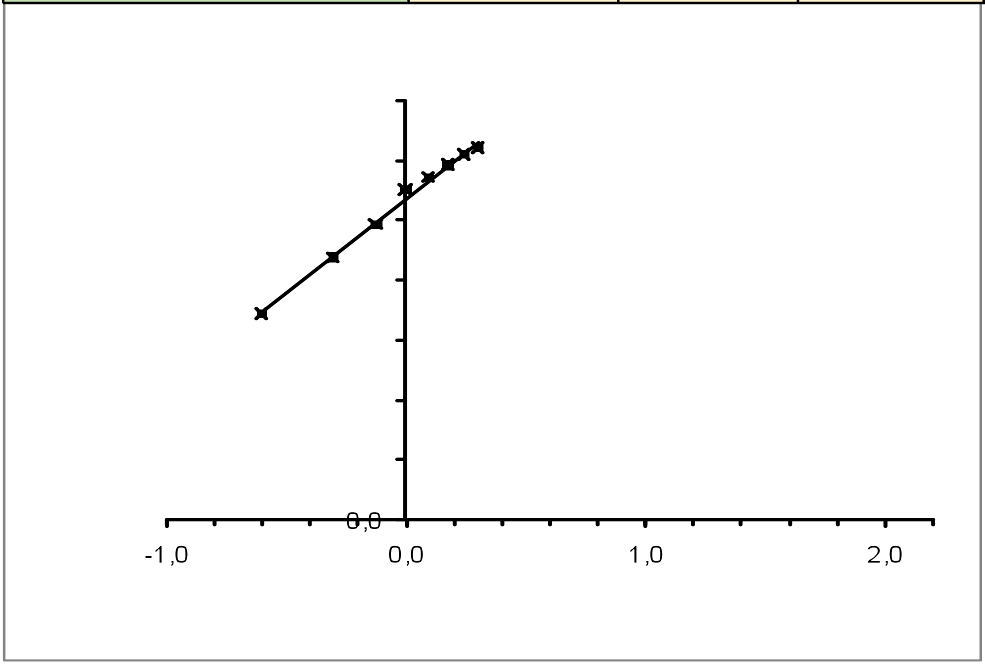


**Figure S15.** 3 % w/v κ-C:gelatin (1:1 mass ratio) hydrogels . Korsmeyer-Peppas model at pH 1.2.

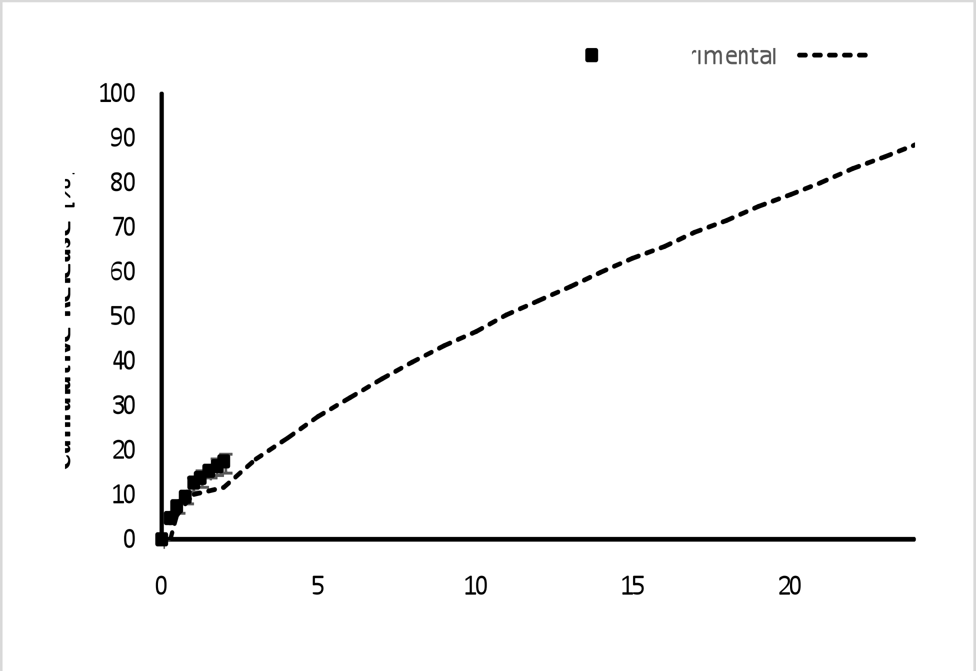


| y = y∞ * a * (1-exp(-(kt)^b)) | y∞ | a | k | b |
|---|---|---|---|---|
| | -0,00471097 | 0,005 | -2,94647E+24 | 0,05 |

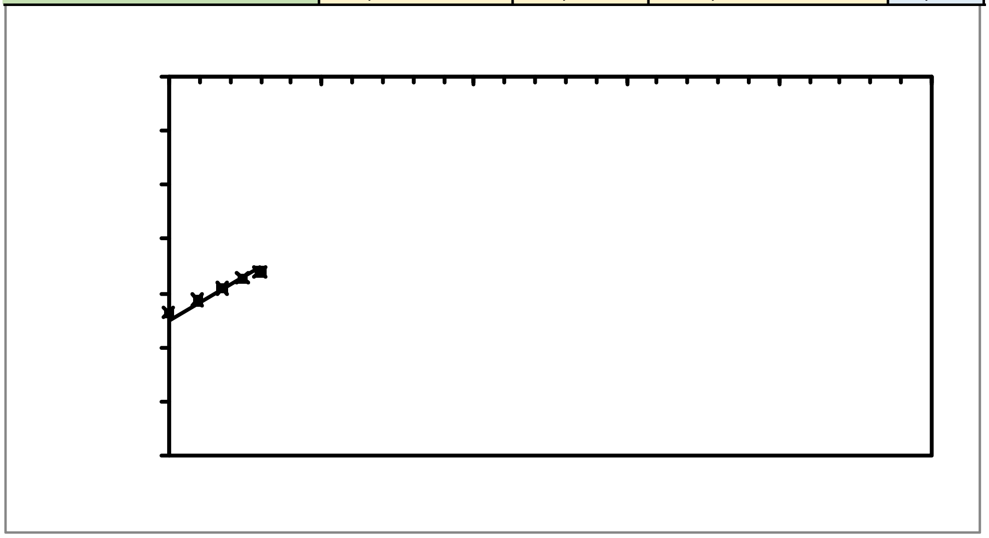

**Figure S16.** 3 % w/v κ-C:gelatin (1:1 mass ratio) hydrogels . Webull model at pH 1.2.

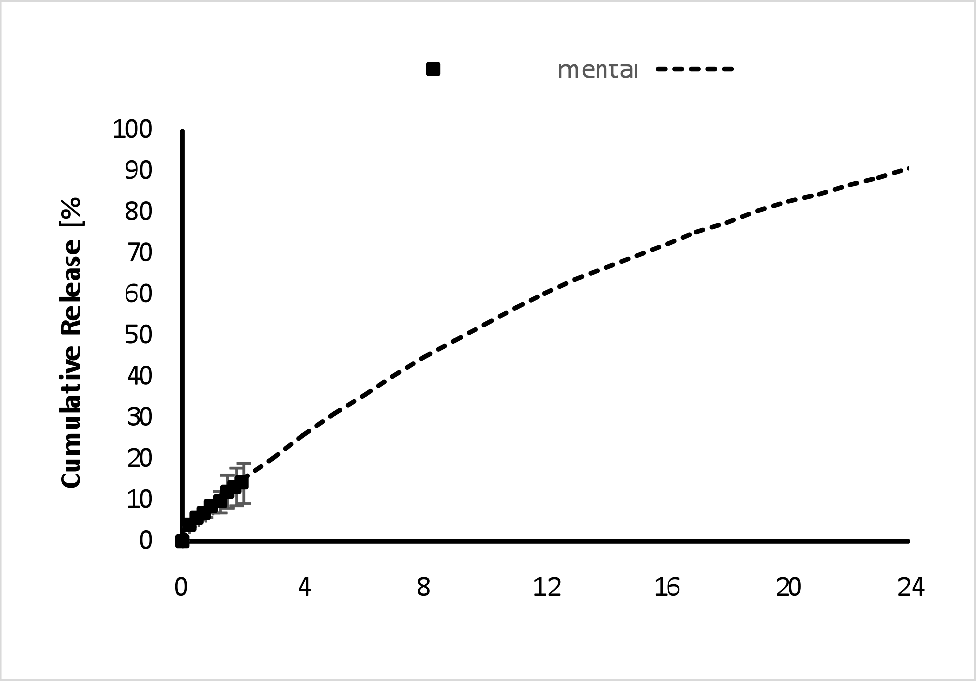


| y = y0 + A * exp(R0 * x) | y0 | A | R0 |
|---|---|---|---|
| | 124,2026331 | -122,07231 | -0,053555867 |

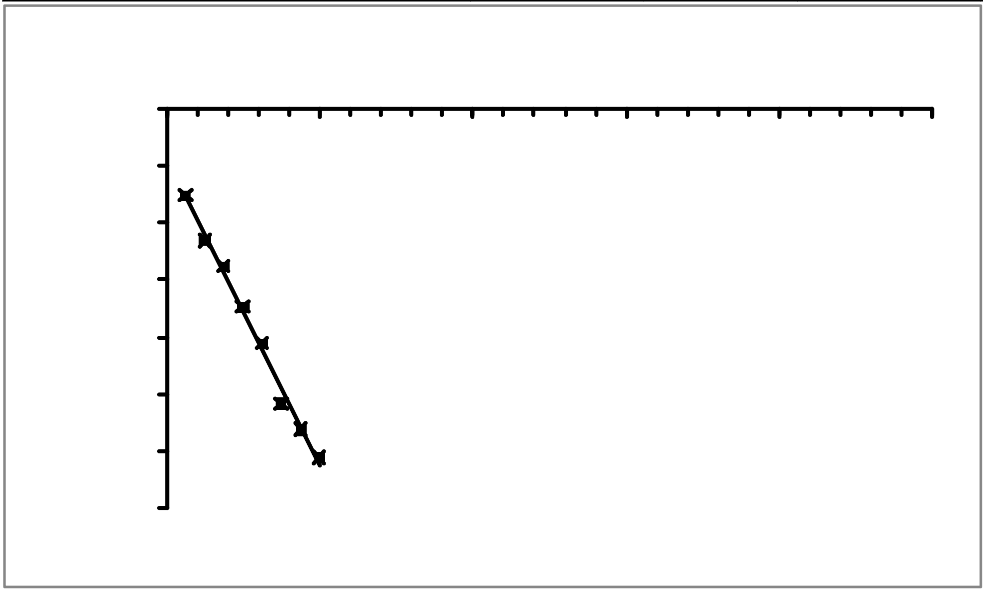

**Figure S17.** 4 % w/v κ-C:gelatin (1:1 mass ratio) hydrogels . First order kinetic model at pH 1.2.

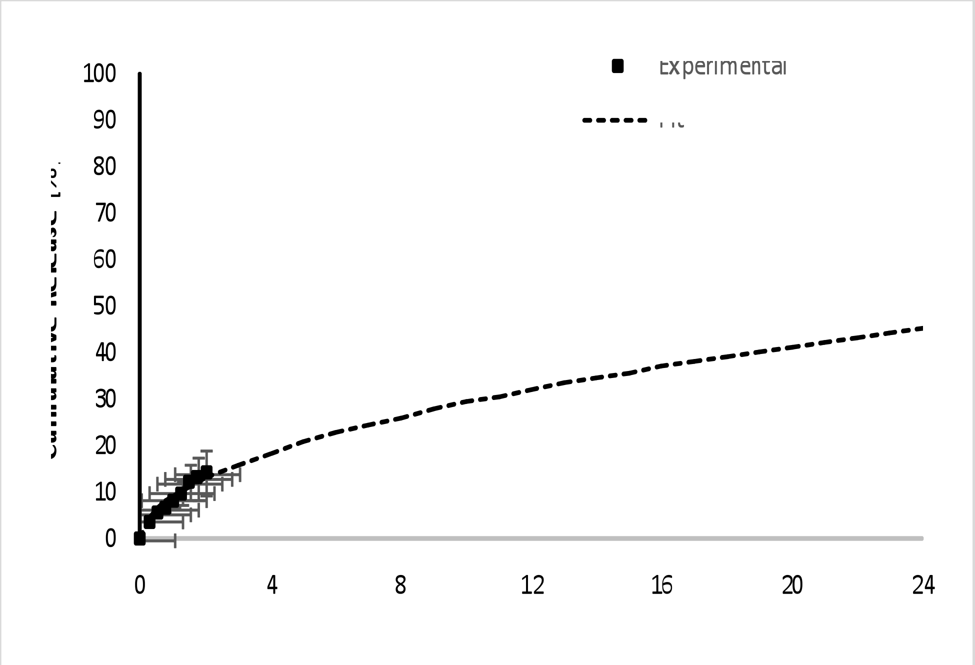


| y = y∞ * k * √t | y∞ | k |
|---|---|---|
| | 43,0342093 | 0,21519152 |

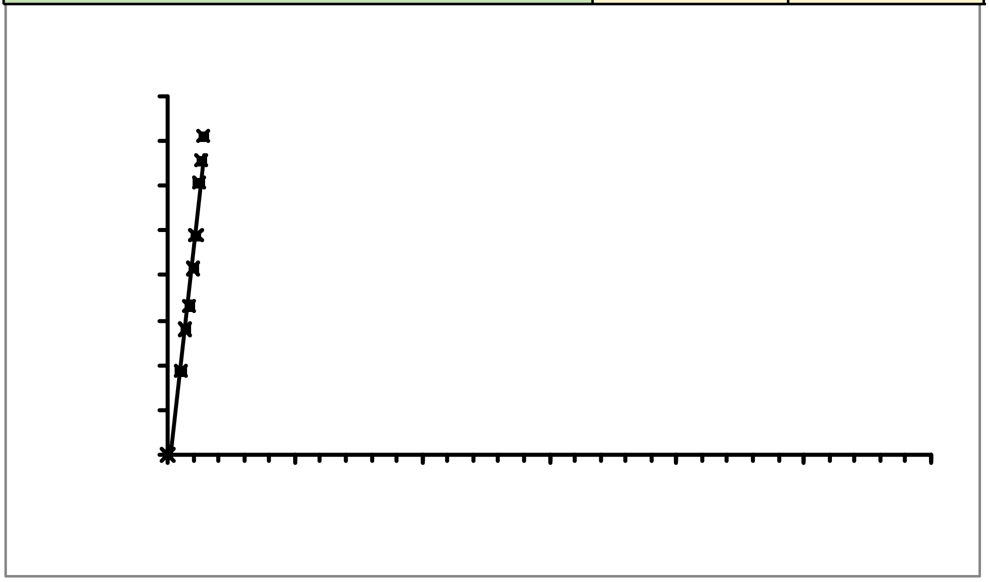

**Figure S18.** 4 % w/v κ-C:gelatin (1:1 mass ratio) hydrogels . Higuchi model at pH 1.2.

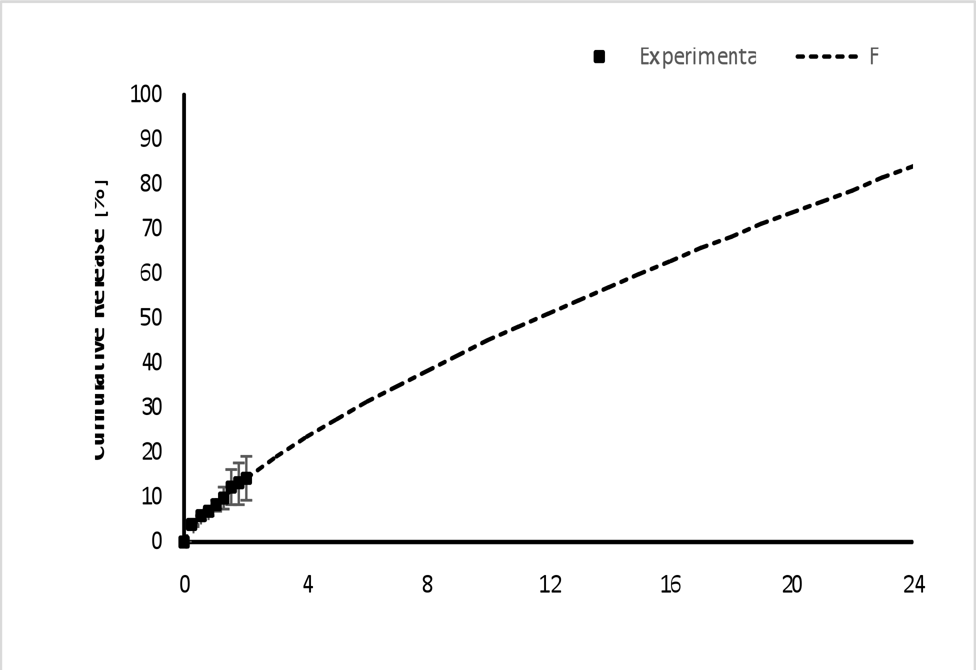


| $y = y_\infty * k * t^n$ | $y_\infty$ | k | n |
|---|---|---|---|
| | 3,059859444 | 2,850225979 | 0,711737189 |

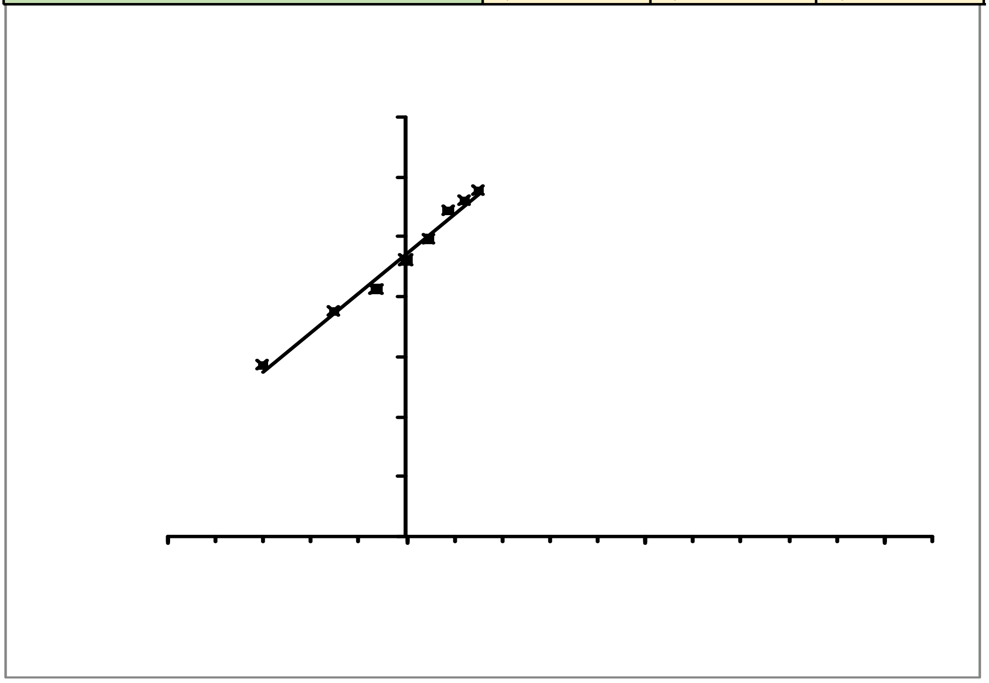

**Figure S19.** 4 % w/v κ-C:gelatin (1:1 mass ratio) hydrogels. Korsmeyer-Peppas model at pH 1.2.

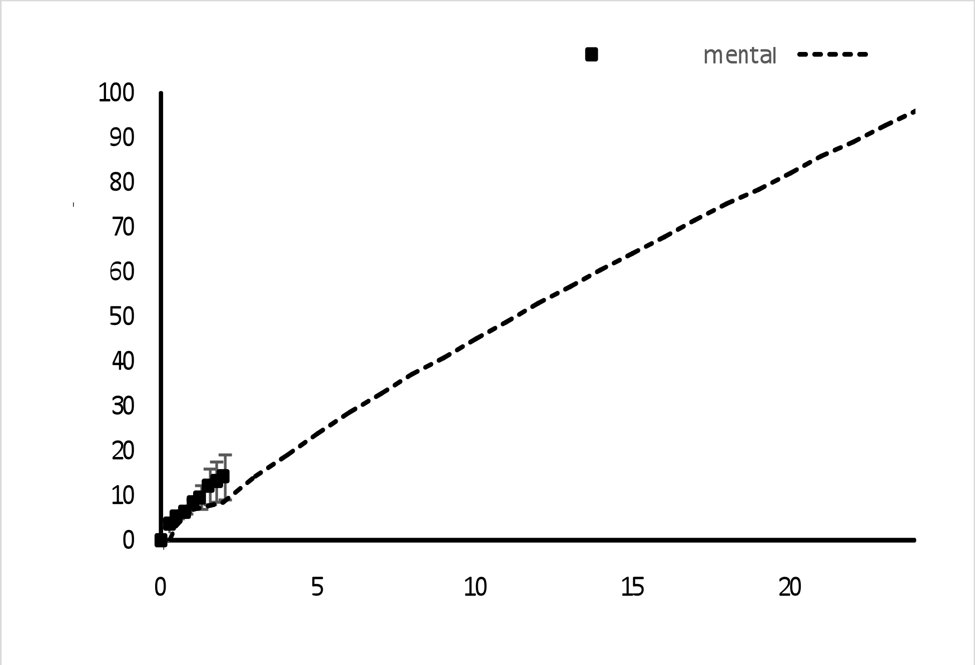


| $y = y\infty * a * (1-exp(-(kt)^b)$ | y∞ | a | k | b |
|---|---|---|---|---|
| | -0,00163361 | 0,002 | -2,94647E+24 | 0,05 |

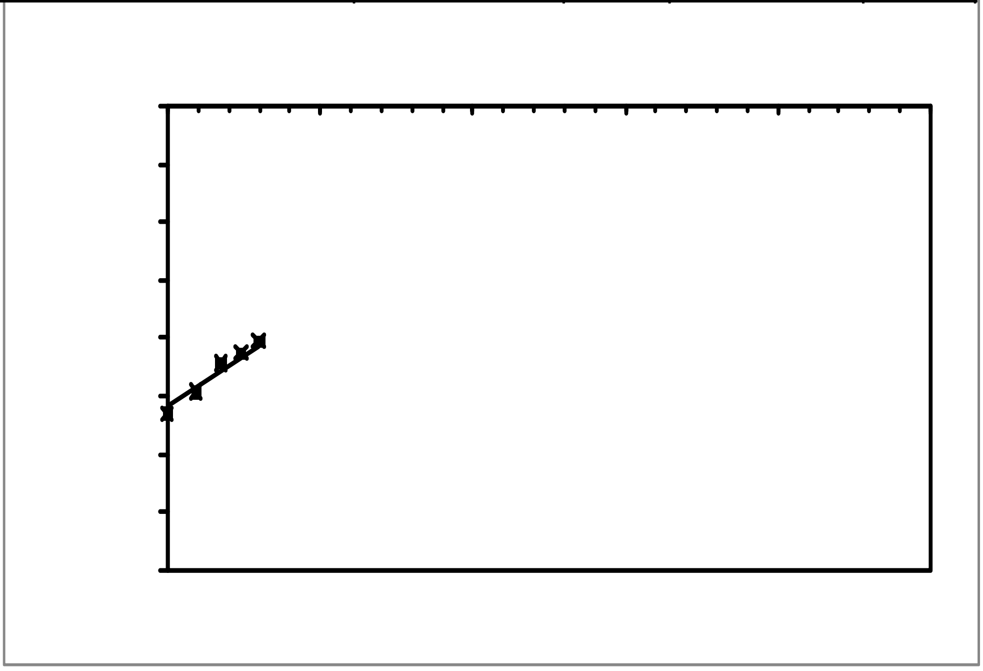

**Figure S20.** 4 % w/v κ-C:gelatin (1:1 mass ratio) hydrogels . Weibull model at pH 1.2.

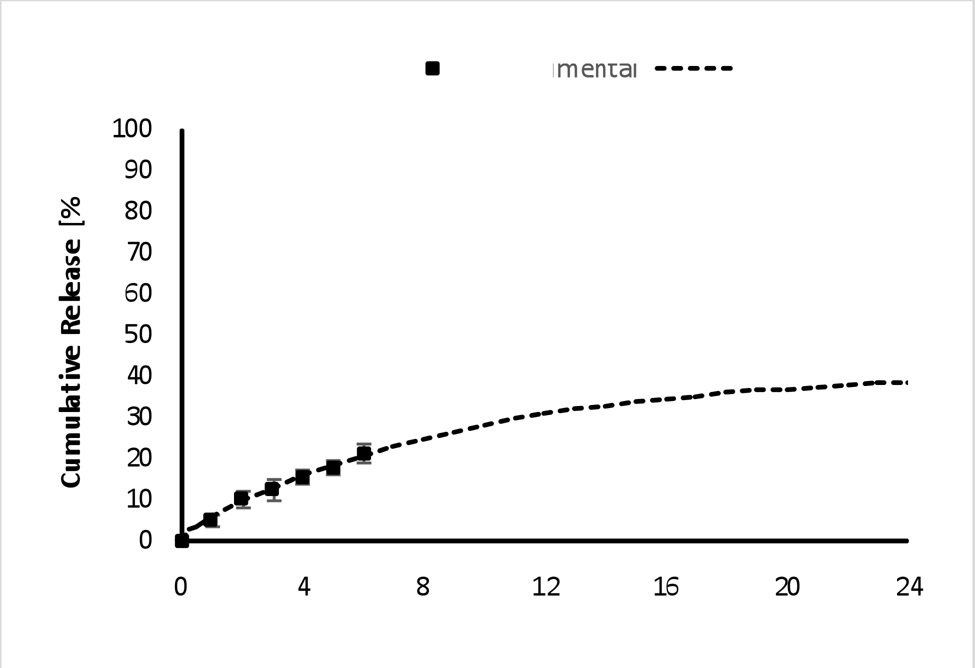


| y = y0 + A * exp(R0 * x) | y0 | A | R0 |
|---|---|---|---|
| | 41,17113248 | -39,719648 | -0,1110271 |

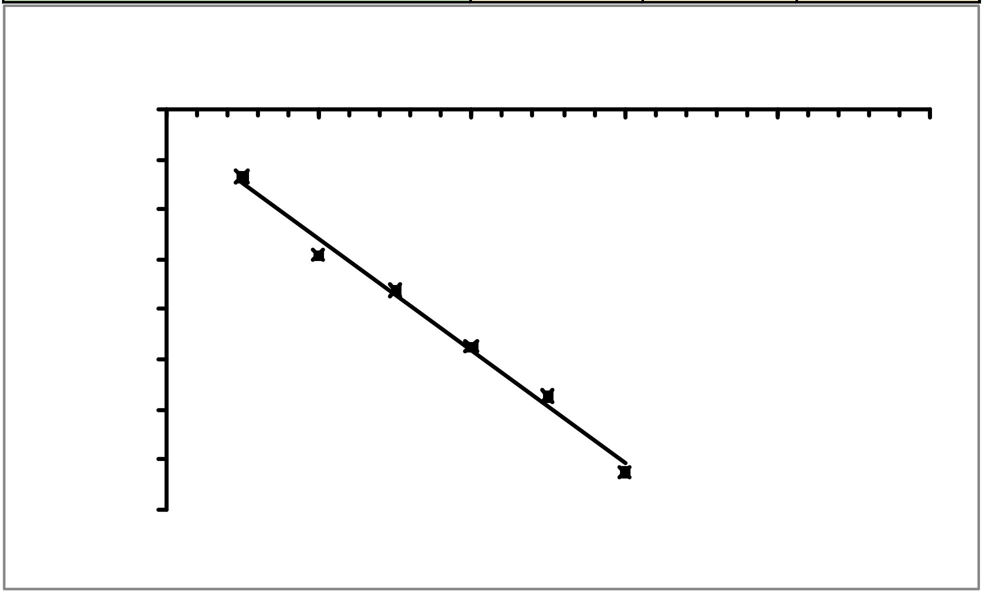

**Figure S21.** 2 % w/v κ-C:gelatin (1:1 mass ratio) hydrogels. First order kinetic model at pH 6.8.

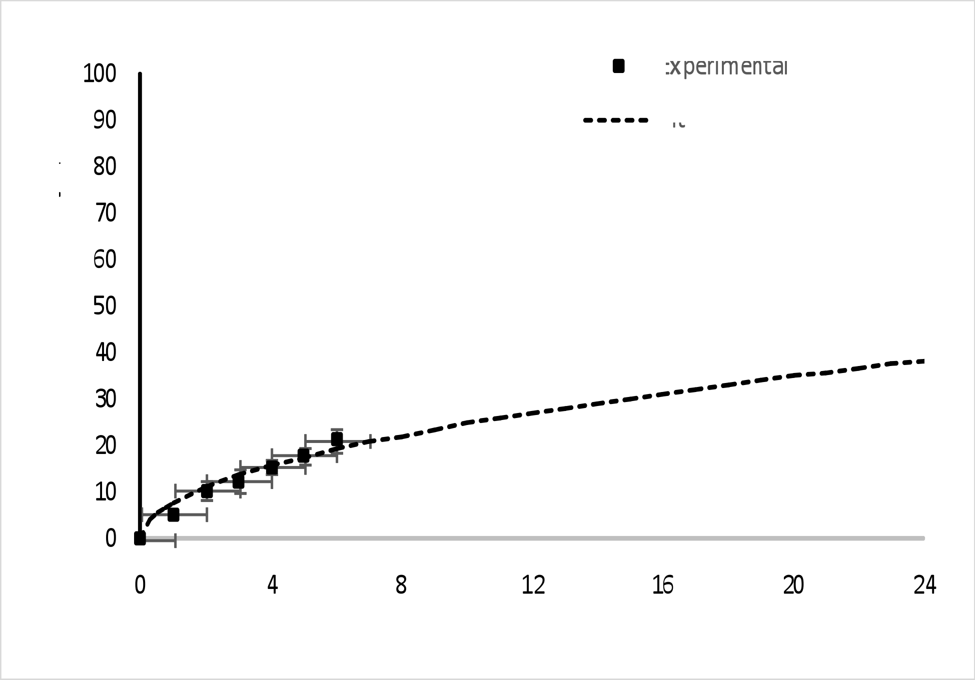


| $y = y_\infty * k * \sqrt{t}$ | $y_\infty$ | k |
|---|---|---|
| | 43,0326545 | 0,18174104 |

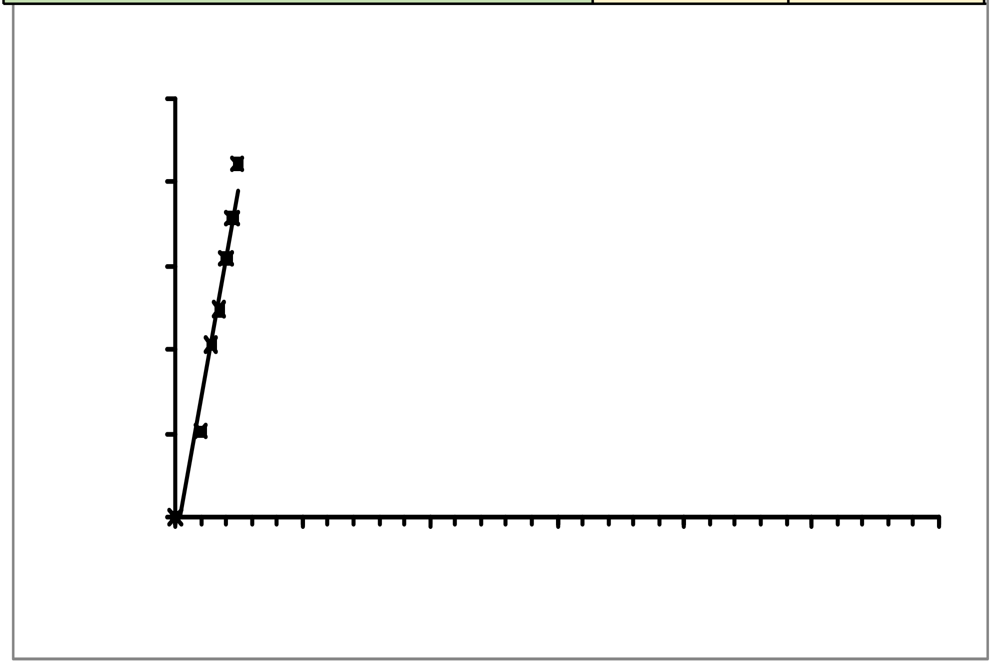

**Figure S22.** 2 % w/v κ-C:gelatin (1:1 mass ratio) hydrogels. Higuchi model at pH 6.8.

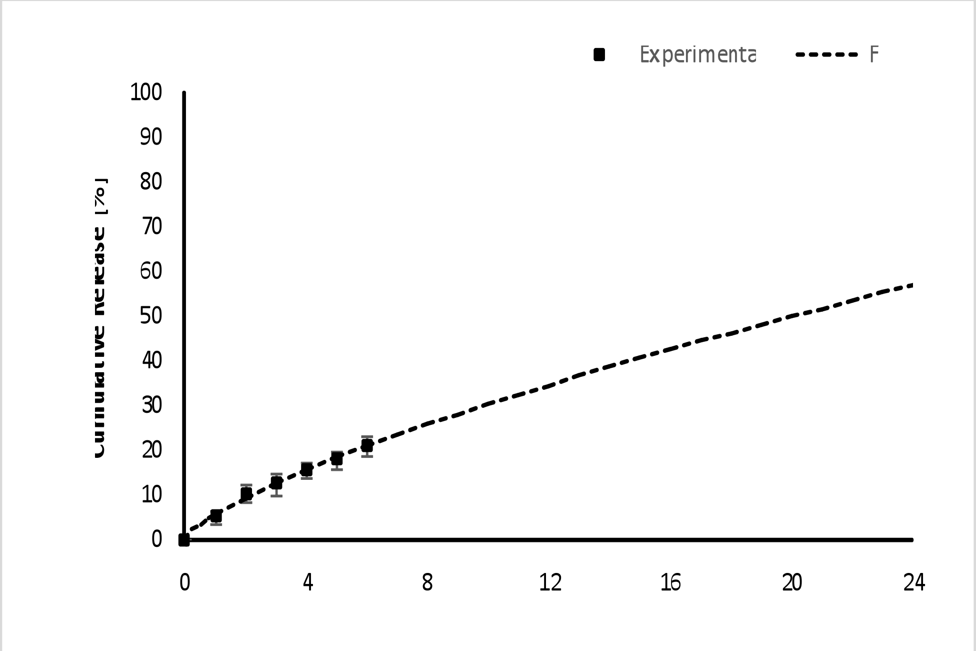


| $y = y\infty * k * t^n$ | y∞ | k | n |
|---|---|---|---|
| | 2,590177178 | 2,207520646 | 0,723063491 |

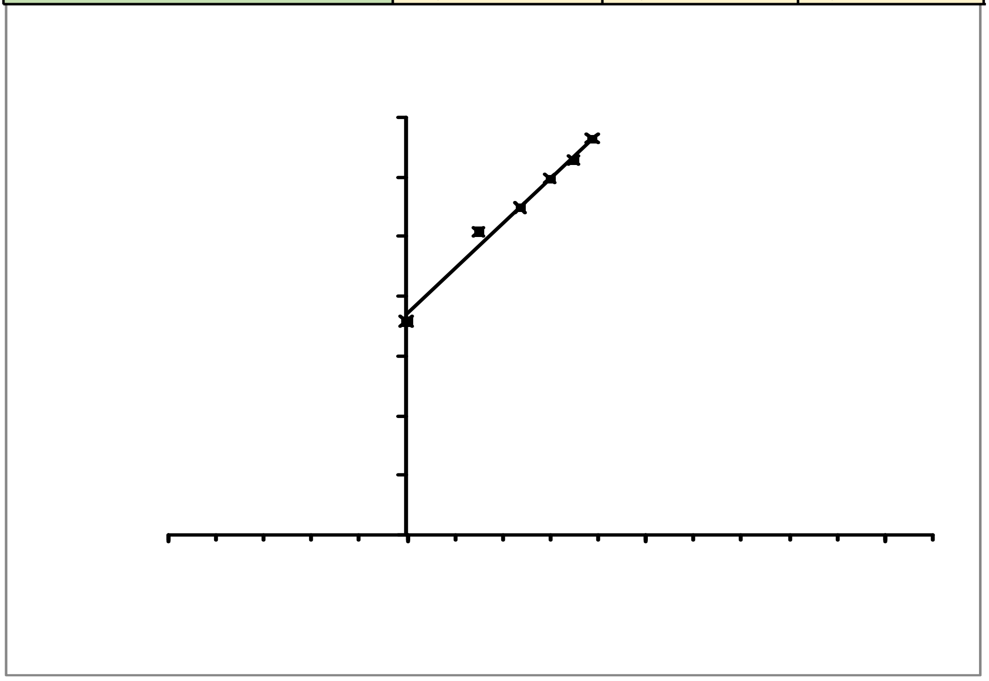

**Figure S23.** 2 % w/v κ-C:gelatin (1:1 mass ratio) hydrogels. Korsmeyer-Peppas model at pH 6.8.

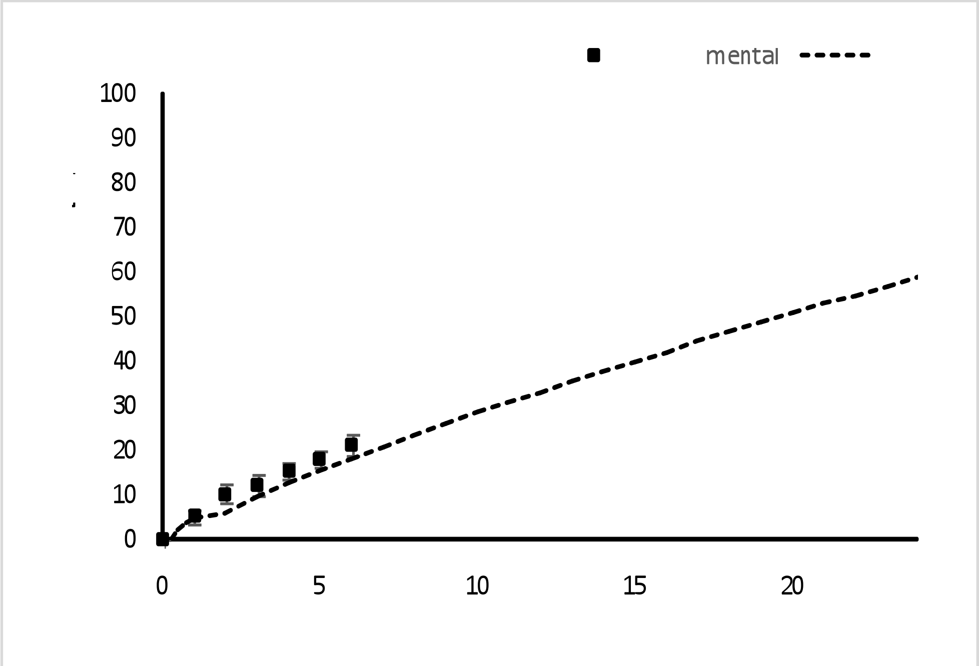


| $y = y\infty * a * (1\text{-}exp(\text{-}(kt)^{b})$ | y∞ | a | k | b |
|---|---|---|---|---|
| | -0,00312707 | 0,001 | -2,94647E+24 | 0,05 |

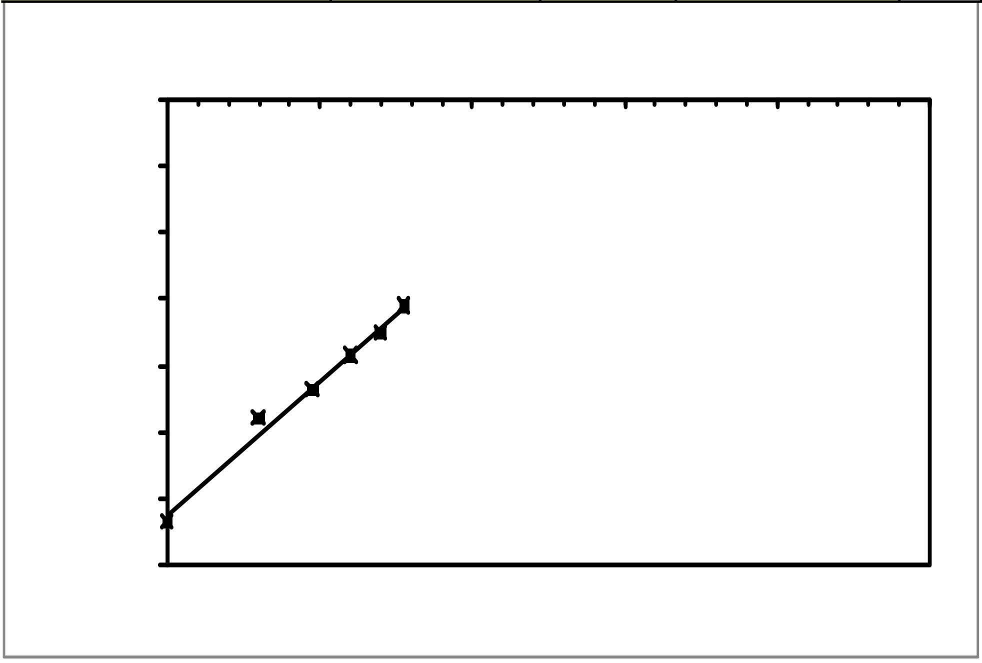

**Figure S24.** 2 % w/v κ-C:gelatin (1:1 mass ratio) hydrogels. Weibull model at pH 6.8.

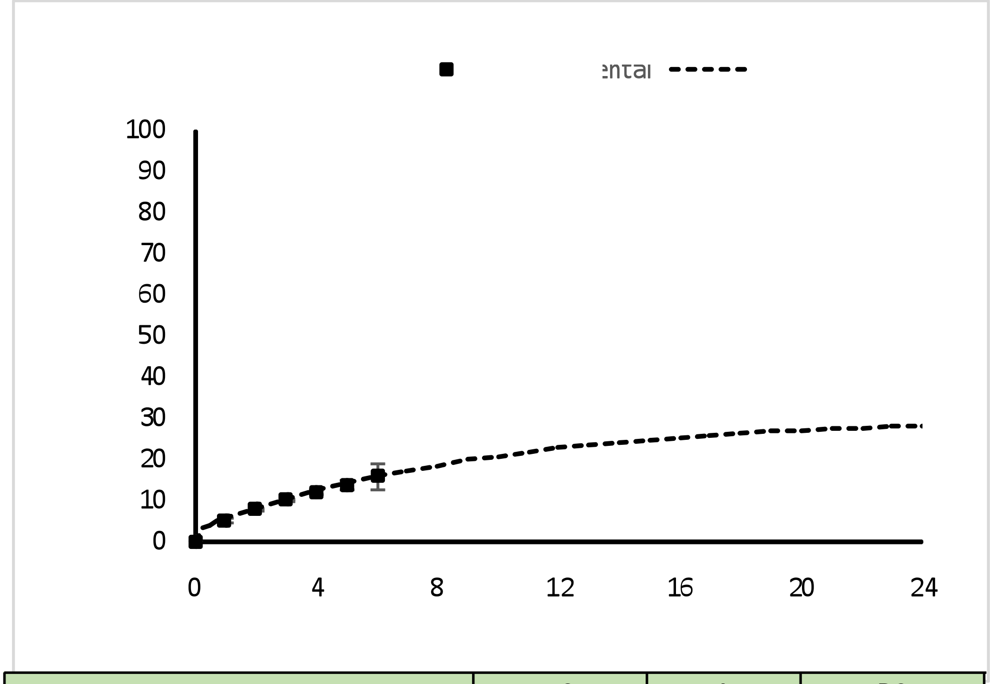


| y = y0 + A * exp(R0 * x) | y0 | A | R0 |
|---|---|---|---|
| | 30,41499475 | -27,764555 | -0,106231109 |

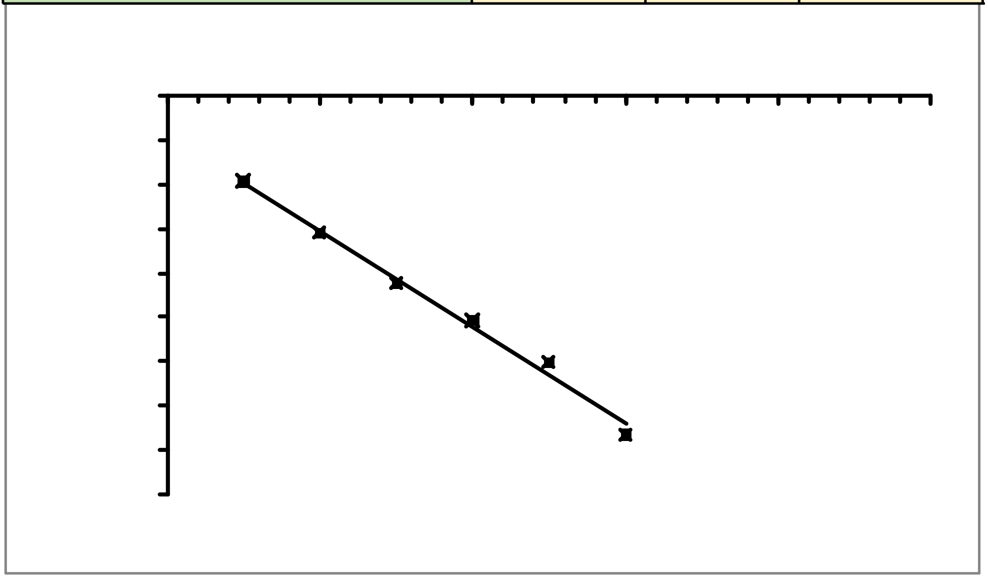

**Figure S25.** 3 % w/v κ-C:gelatin (1:1 mass ratio) hydrogels . First order kinetic model at pH 6.8.

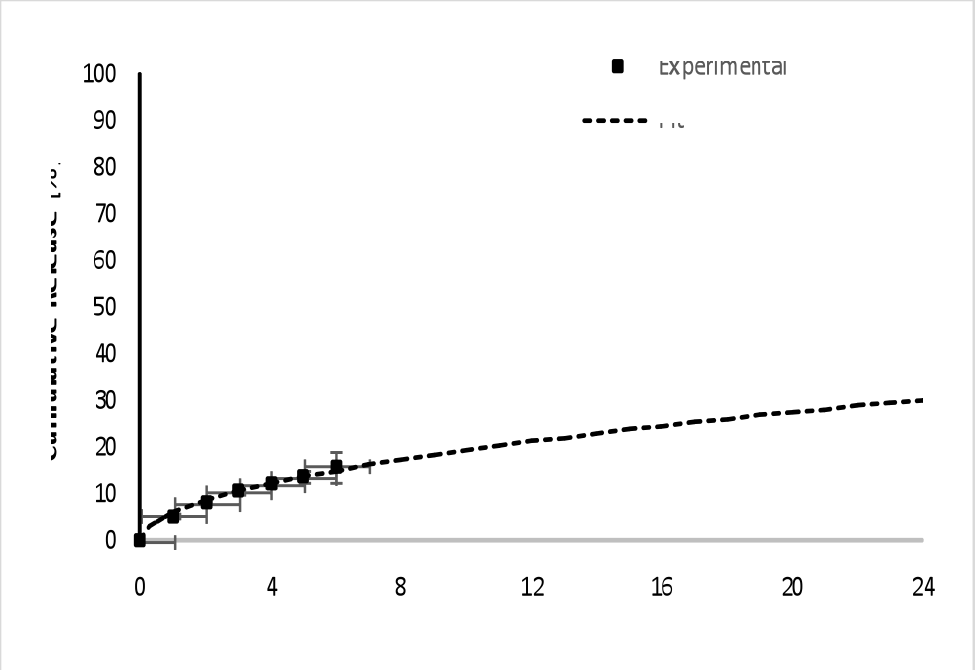


| y = y∞ * k * √t | y∞ | k |
|---|---|---|
| | 43,0331782 | 0,1427619 |

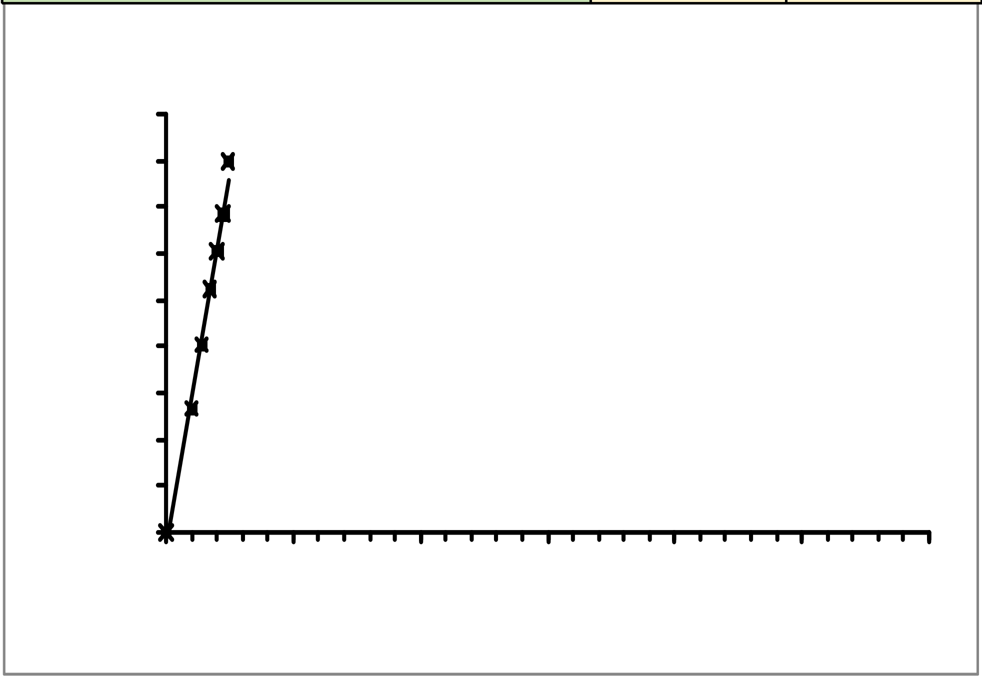

**Figure S26.** 3 % w/v κ-C:gelatin (1:1 mass ratio) hydrogels . Higuchi model at pH 6.8.

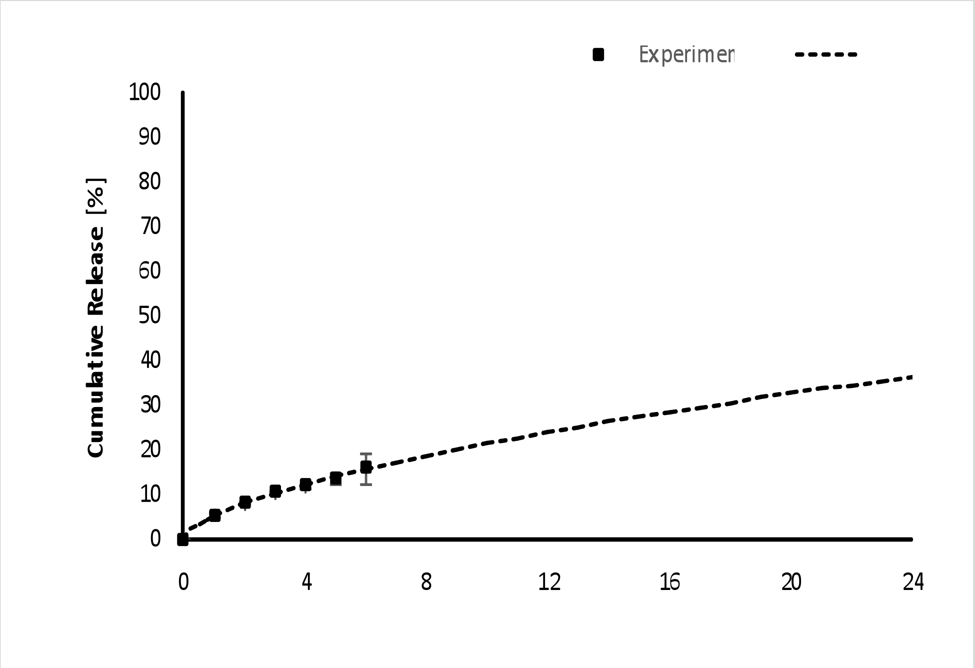


| $y = y\infty * k * t^n$ | y∞ | k | n |
|---|---|---|---|
| | 2,470690501 | 2,144964828 | 0,606084034 |

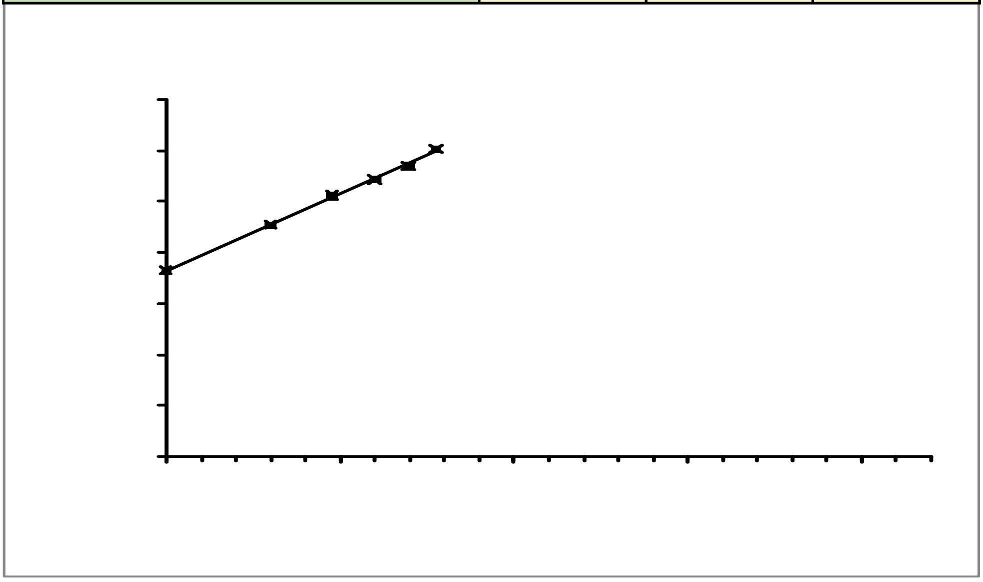

**Figure S27.** 3 % w/v κ-C:gelatin (1:1 mass ratio) hydrogels . Korsmeyer-Peppas model at pH 6.8.

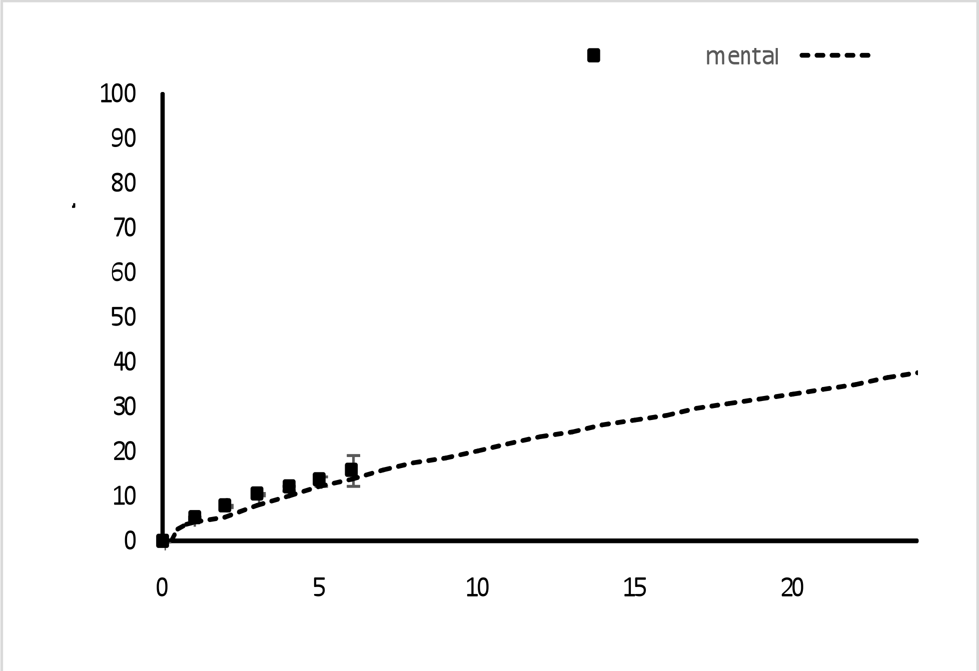


| $y = y∞ * a * (1\text{-}exp(-(kt)^b)$ | y∞ | a | k | b |
|---|---|---|---|---|
| | 0,000802502 | -0,019 | -2,94647E+24 | 0,05 |

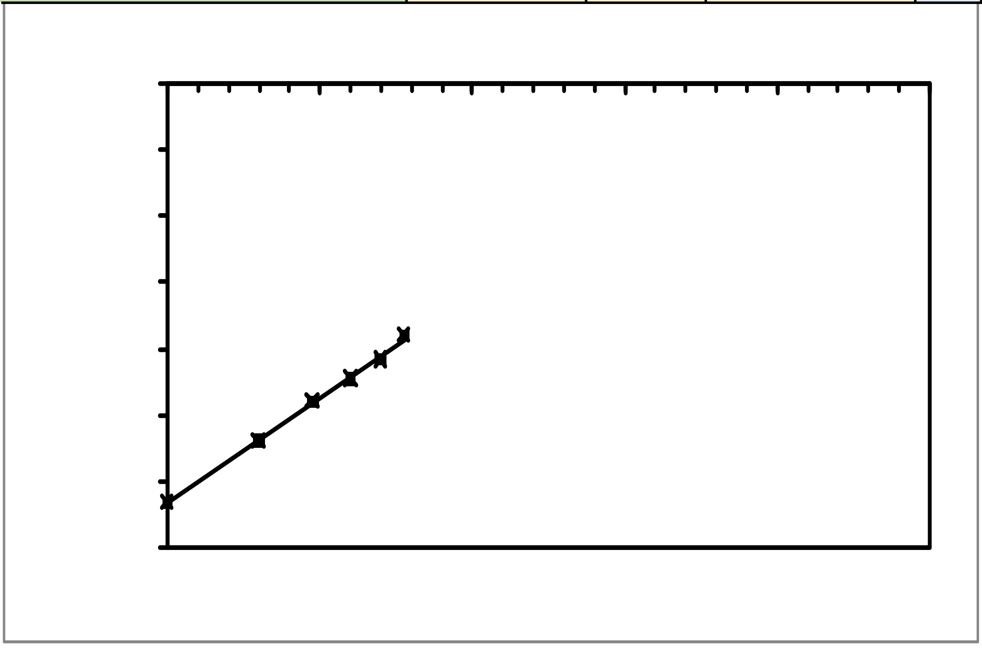

**Figure S28.** 3 % w/v κ-C:gelatin (1:1 mass ratio) hydrogels . Weibull model at pH 6.8.

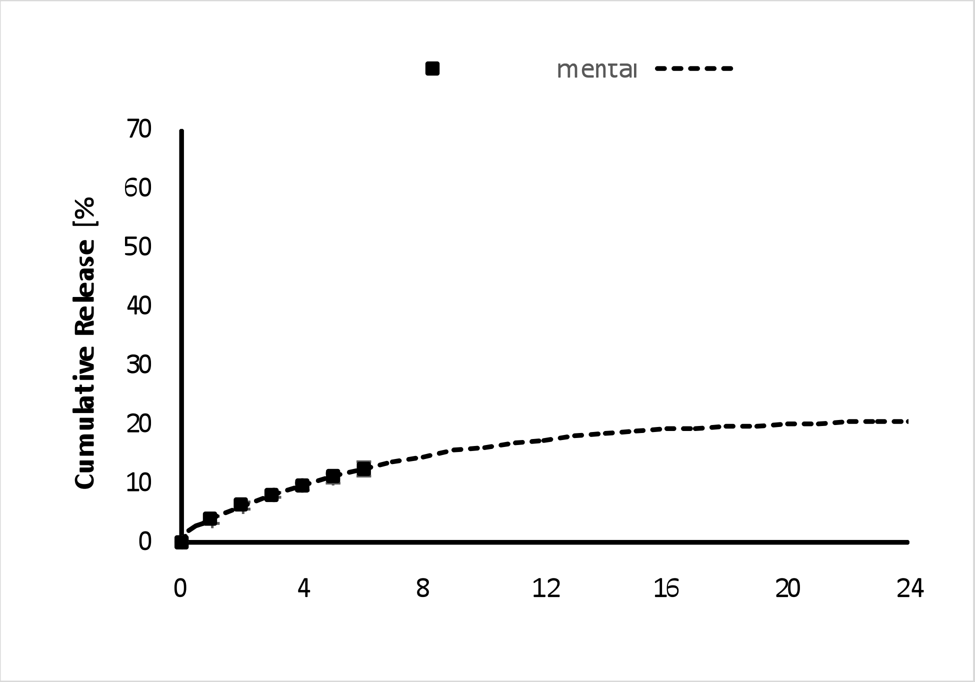


| y = y0 + A * exp(R0 * x) | y0 | A | R0 |
|---|---|---|---|
| | 21,2557884 | -19,938854 | -0,136578669 |

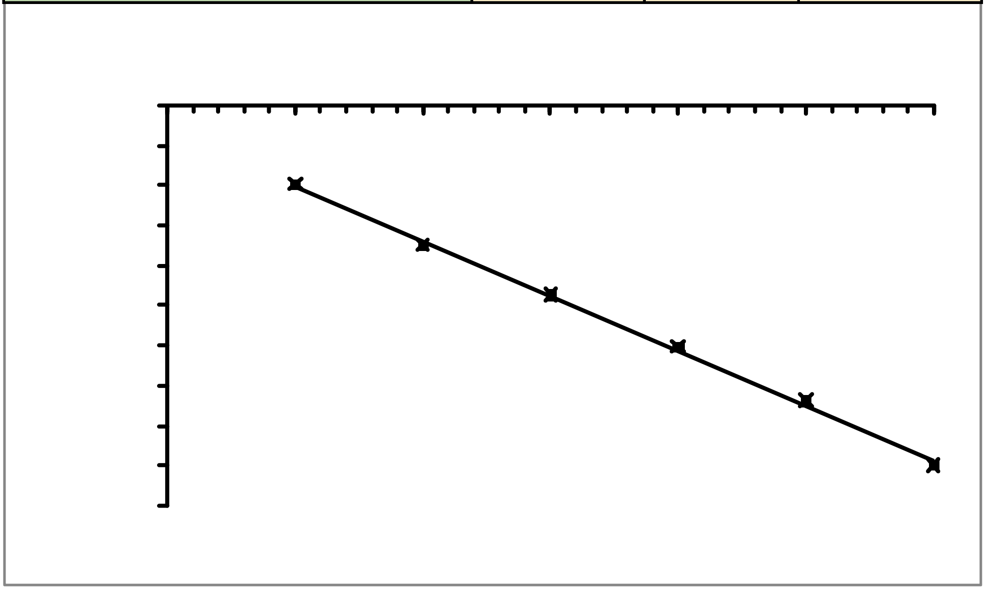

**Figure S29.** 4 % w/v κ-C:gelatin (1:1 mass ratio) hydrogels. First order kinetic model at pH 6.8.

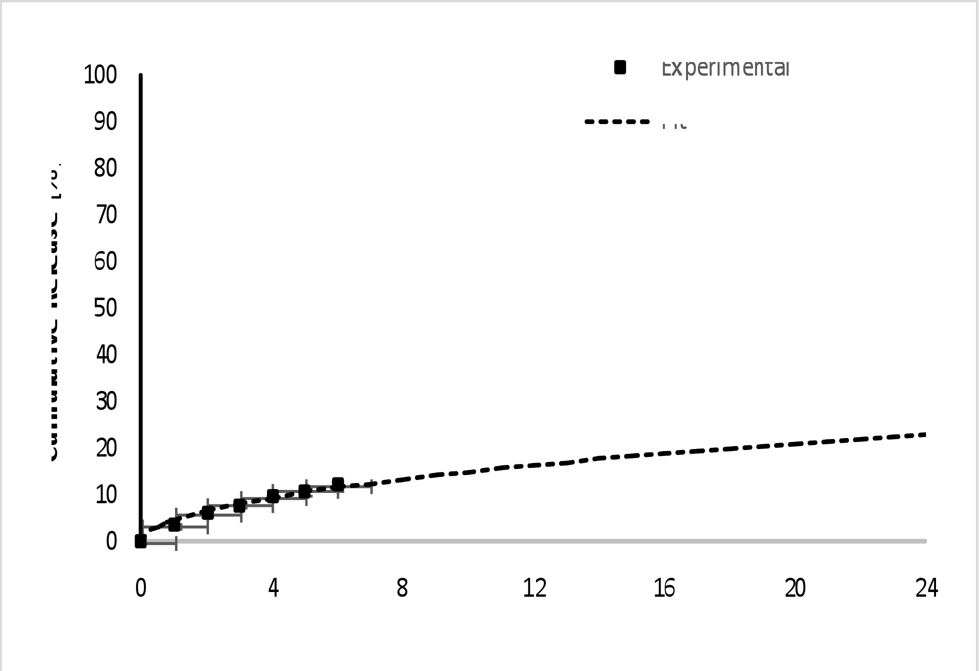


| $y = y\infty * k * \sqrt{t}$ | y∞ | k |
|---|---|---|
| | 43,0338816 | 0,10942306 |

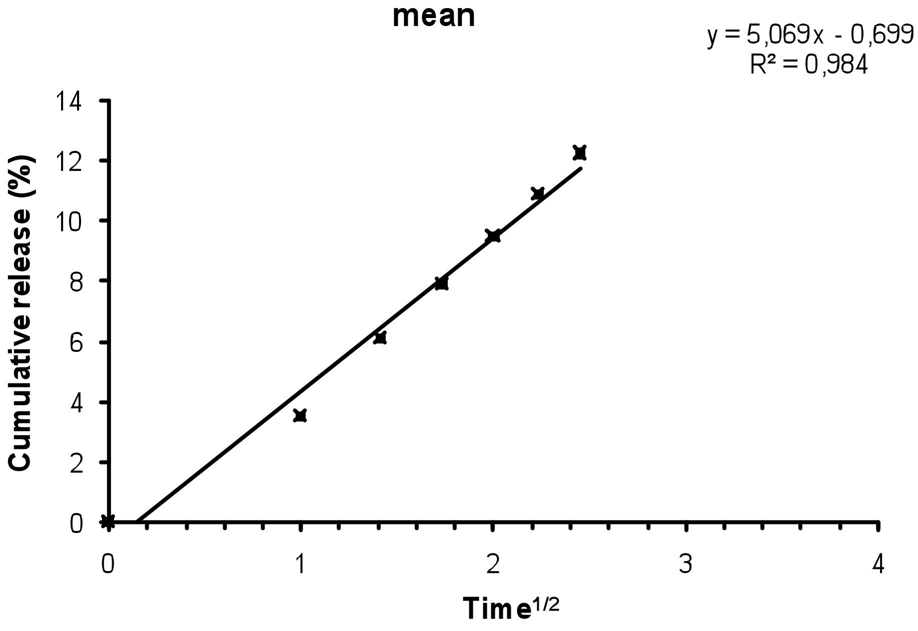


**Figure S30.** 4 % w/v κ-C:gelatin (1:1 mass ratio) hydrogels. Higuchi model at pH 6.8.

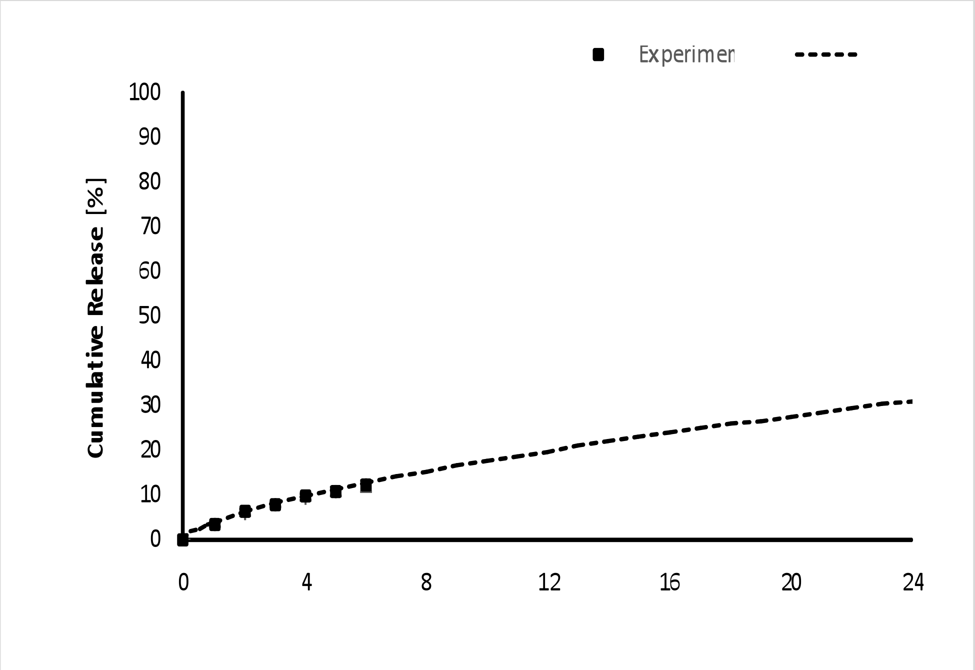


| $y = y_\infty * k * t^n$ | y∞ | k | n |
|---|---|---|---|
| | 3,67380291 | 1,062078706 | 0,652022313 |

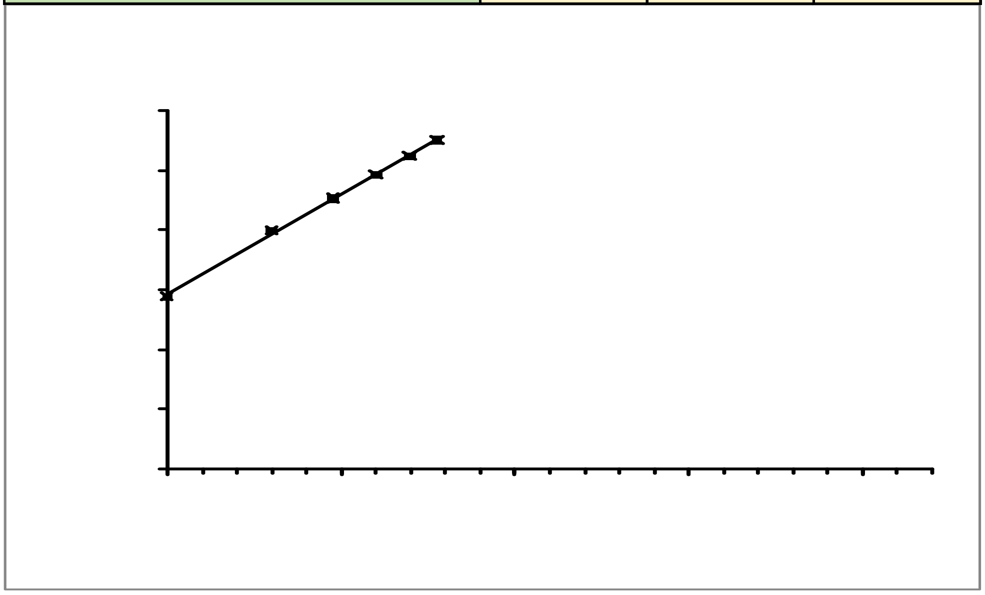

**Figure S31.** 4 % w/v κ-C:gelatin (1:1 mass ratio) hydrogels. Korsmeyer-Peppas model at pH 6.8.

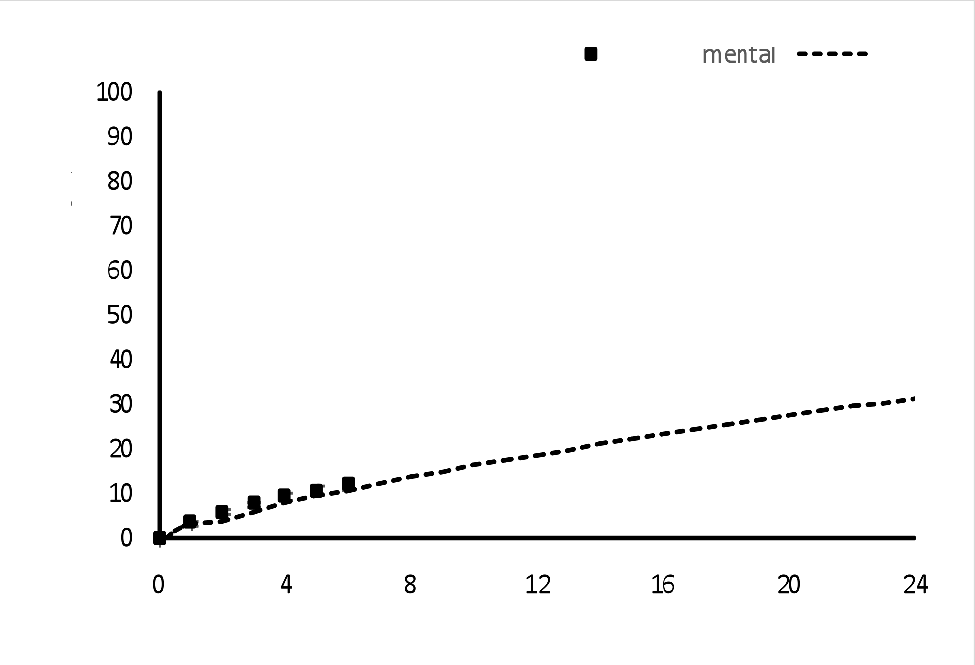


| | y∞ | a | k | b |
|---|---|---|---|---|
| $y = y_\infty * a * (1-\exp(-(kt)^b)$ | 0,00261181 | -0,002 | -2,94647E+24 | 0,05 |

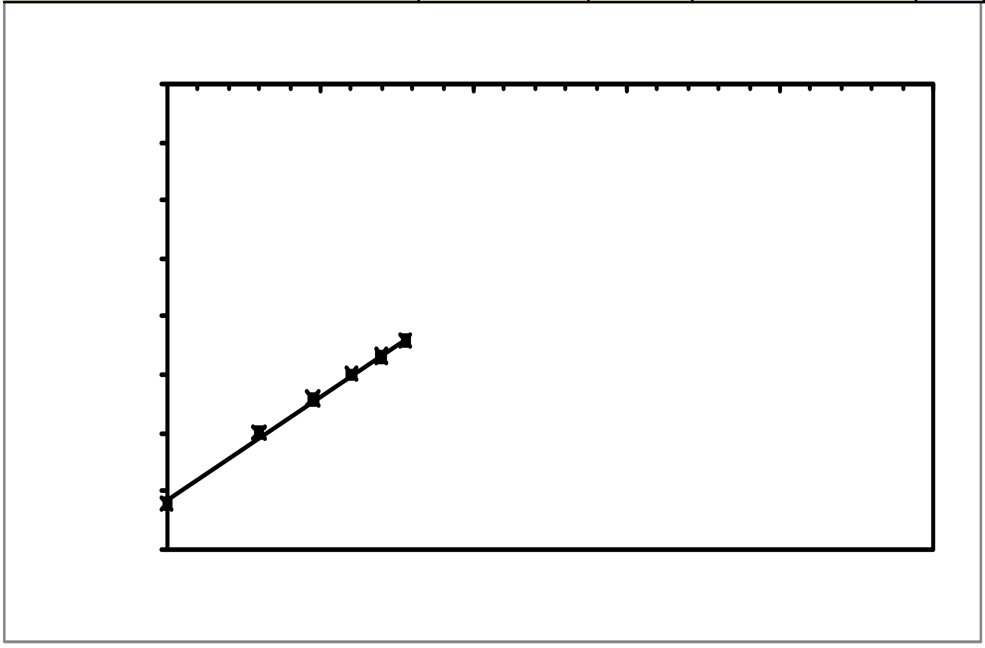

**Figure S32.** 4 % w/v κ-C:gelatin (1:1 mass ratio) hydrogels. Weibull model at pH 6.8.

## 6. Photoisomerization experiments

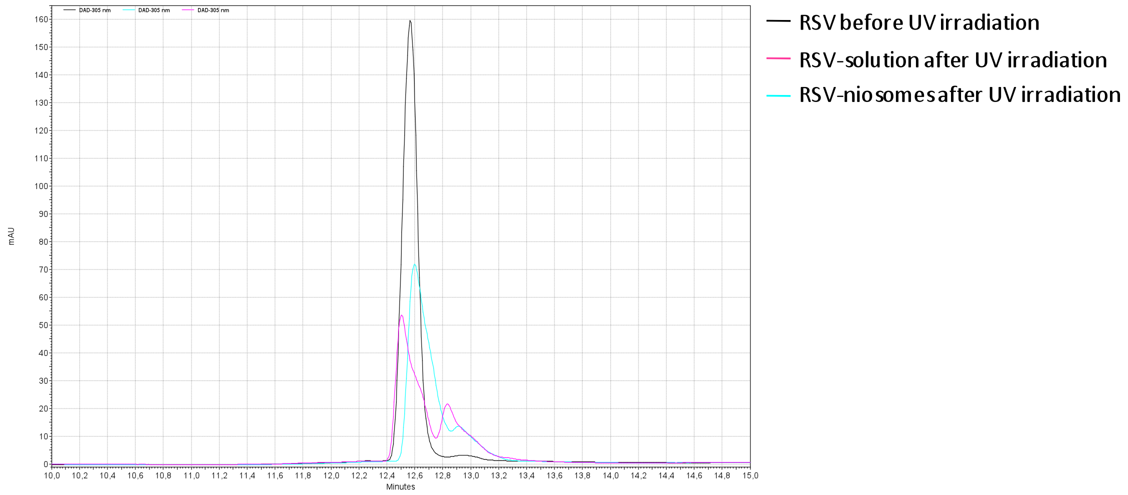


**Figure S33.** Chromatograms of RSV before (black line) and after UV irradiation (pink and light blue lines for RSV ethanolic control solution and RSV in niosomes, respectively).

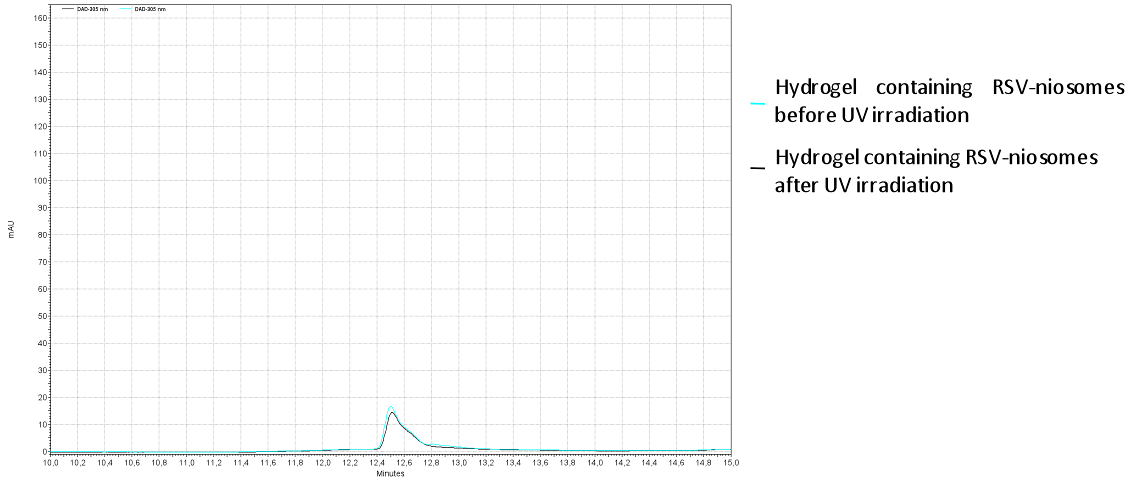


**Figure S34.** Chromatograms of RSV in niosomes encapsulated in hydrogel before and after UV-irradiation (light blue and black lines, respectively).

**Table S8.** Molar ratio of *trans*-RSV remaining in the sample after UV irradiation.

| Sample | *trans*-RSV remaining |
|---|---|
| Ethanolic control solution | 0,26 ± 0,03 |
| Niosomes | 0,70 ± 0,07 |
| Niosomes entrapped hydrogel | 0,89 ± 0,03 |
